# Optimizing carbohydrate quality: a path to better health for women with PCOS

**DOI:** 10.3389/fnut.2025.1578459

**Published:** 2025-06-18

**Authors:** Li Zhang, Yuxin Jin, Aili Yang, Xinwen Yu, Yi Li, Xin Wang, Chunni Heng, Biao Qi, Bin Gao, Guohong Zhao

**Affiliations:** ^1^Department of Endocrinology, Tangdu Hospital, Air Force Medical University, Xi’an, Shaanxi, China; ^2^Department of Gynecology and Obstetrics, Tangdu Hospital, Air Force Medical University, Xi’an, Shaanxi, China; ^3^The Key Laboratory of Biomedical Information Engineering of Ministry of Education, School of Life Sciences and Technology, Xi’an Jiaotong University, Xi’an, China; ^4^Bioinspired Engineering and Biomechanics Center (BEBC), Xi’an Jiaotong University, Xi’an, China

**Keywords:** polycystic ovary syndrome (PCOS), carbohydrate quality, glycolipid metabolism, sex hormones, meta-analysis

## Abstract

**Introduction:**

The rising global prevalence of polycystic ovary syndrome (PCOS) poses a significant threat to women’s metabolic and reproductive health. The carbohydrate quality—particularly dietary fiber, glycemic index (GI), and glycemic load (GL)—in addressing metabolic and reproductive abnormalities remains debated due to the condition’s heterogeneity. “The ongoing debate regarding PCOS arises from its complexity and heterogeneity, including variations in clinical symptoms, underlying causes, and treatment responses.” This study aimed to perform a meta-analysis of randomized clinical trials to examine the effects of high fiber and low glycemic index (LGI)/low glycemic load (LGL) dietary interventions on metabolic parameters in women with PCOS.

**Methods:**

A systematic literature search was conducted using PubMed, EMBASE, the Cochrane Library, Web of Science, Ovid MEDLINE, and Scopus to identify eligible studies. The outcomes were reported as standardized mean differences (SMD) with 95% confidence intervals (CI). Heterogeneity among studies was evaluated using the chi-square test and the I^2^ statistic.

**Results:**

The study showed high dietary fiber and LGI significantly reduced fasting glucose and insulin resistance. Both high fiber and the LGI diet significantly reduced triglycerides and Low-density lipoprotein cholesterol (LDL-C), with fiber also increasing High-density lipoprotein cholesterol (HDL-C). High-fiber and LGI diets increased Sex Hormone-Binding Globulin (SHBG) and reduced Free androgen index (FAI).

**Discussion:**

This meta-analysis highlights the significant benefits of optimizing dietary carbohydrate quality on glycolipid metabolism, sex hormone levels, and weight in women with PCOS. While further high-quality studies are needed, the findings suggest that dietary fiber and LGI/LGL consumption have distinct effects on metabolic parameters. Therefore, treatment strategies should incorporate personalized dietary interventions tailored to the specific needs of women with PCOS within a shared decision-making framework.

**Systematic review registration:**

www.crd.york.ac.uk, identifier PROSPERO CRD42024579681.

## Graphical Abstract

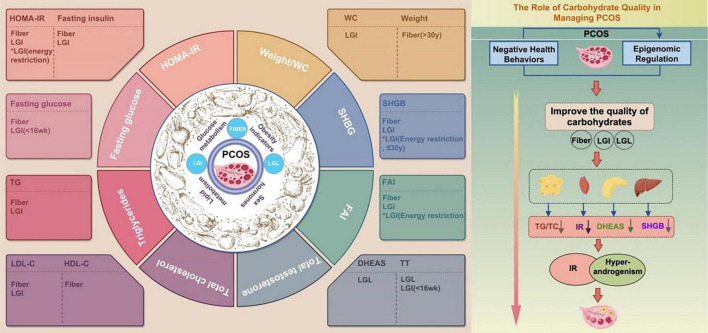

## 1 Introduction

Polycystic ovary syndrome (PCOS) is a common endocrine disorder that is estimated to affect approximately 15–20% of women via the Rotterdam criteria (1). PCOS is highly heterogeneous, which is due mainly to its reproductive, metabolic, and endocrine abnormalities. In addition to hyperandrogenaemia and ovulation disorders, women with PCOS often present with metabolic abnormalities such as abnormal glycolipid metabolism, obesity, and insulin resistance, which eventually lead to an increased risk of type 2 diabetes and coronary heart disease (2–5). The pathogenesis of PCOS is complex and involves various factors, such as genetics, the environment and lifestyle. Among them, lifestyle intervention, especially dietary intervention, is considered an important means to manage the symptoms and complications of PCOS (6, 7). However, owing to the heterogeneity and individual differences in PCOS, the optimal dietary regimen has not yet been determined. Nevertheless, it has become increasingly clear that the dietary strategy for the successful management of PCOS should be “beyond calories” and focus on dietary quality (7). Moreover, follow-up studies have shown that different dietary patterns have different effects on the metabolic and reproductive characteristics of individuals with PCOS (8). Therefore, developing individual dietary guidelines for PCOS patients to fully consider the needs of their different metabolic abnormalities is particularly important. Compared with reproductive system diseases, PCOS is increasingly considered a metabolic disease, and comprehensive management of the long-term metabolic consequences is needed (9). Among the studies on interventions involving different dietary patterns in individuals with PCOS, the studies involving “carbohydrates” were the most extensive (3–7, 10). Refined carbohydrates, high glycemic index (GI) and glycemic load (GL), and a low-fiber diet are associated with PCOS (11–13). A high-fiber diet can significantly improve insulin sensitivity and reduce insulin resistance in PCOS patients (14); at the same time, dietary fiber can weaken energy through energy dilution (15), reduce nutrient absorption (16), suppress appetite (17–19), regulate energy homeostasis (20–22) and alter the gut microbiota (23) and other mechanisms to improve metabolic health and body weight. Compared with refined grains, whole grains (such as oats, brown rice, and whole wheat) are rich in fiber, B vitamins and some trace minerals, such as iron, magnesium, and zinc. The intake of more whole grains helps stabilize blood glucose levels, reduce inflammation, and improve metabolic health (24). Women with PCOS usually overconsume high-GI foods (13), and a low-GI/GL diet has been shown to improve insulin sensitivity, reduce androgen levels, and improve menstrual disorders in PCOS patients (25). However, improvements in specific dietary patterns for different metabolic phenotypes of PCOS also vary. For example, a 24-week low-GI diet intervention study involving obese PCOS women revealed that although the participants’ health-related quality of life improved, the effects of weight loss and metabolic improvement were not significant in some participants (26). Another short-term (4 weeks) low-GL diet study revealed that while this dietary pattern improved self-reported satiety, the improvements in metabolic indicators such as insulin sensitivity and body weight were not significant (27). In summary, as a metabolic disease of PCOS, increasing the quality of carbohydrates can improve the metabolic health and quality of life of PCOS patients to some extent. However, when different carbohydrate quality indicators are used in the face of the heterogeneity of PCOS, the results are still controversial. This further hinders the implementation of individualized dietary management for women with PCOS.

Carbohydrates are a fundamental energy source in various staple plant-based foods. In 2022, the World Health Organization (WHO) highlighted carbohydrate quality—defined by factors such as dietary fiber, whole grain content, free sugar intake, GI, and GL—as a key determinant of diet quality (28). Emerging evidence indicates that prioritizing carbohydrate quality over quantity may significantly reduce the risk of non-communicable diseases (NCDs). Dietary fiber intake significantly impacts metabolic health in women with PCOS by improving insulin sensitivity and regulating blood glucose and lipid levels. Long-term fiber consumption has also been associated with a lower risk of cardiovascular disease and type 2 diabetes. For example, high fiber intake is linked to a 13–33% reduction in the incidence of coronary heart disease, stroke, and colorectal cancer (29, 30). In addition, the effects of fiber should be considered alongside glycemic index (GI) and glycemic load (GL), as low-GI diets are associated with reduced risks of type 2 diabetes and stroke, while high-GI/GL diets may increase metabolic disease risk (2). Therefore, the combined impact of fiber, GI, and GL plays an important role in dietary strategies for PCOS management. Nonetheless, the relationship between GI/GL and health outcomes remains contentious, with some studies questioning the reliability of the GI tract as a predictive marker due to interindividual variability and inconsistent findings in cardiovascular disease intervention studies (31). In conclusion, although carbohydrate quality is closely associated with metabolic disease risk, further research is necessary to elucidate its role in mitigating metabolic disturbances in PCOS patients and to develop tailored dietary recommendations for this population. In this study, we performed a systematic review and meta-analysis on the relationships between the quality of carbohydrates (dietary fiber, whole grains, GI and GL) and various metabolic pathways (such as glycolipid metabolism and sex hormone metabolism) in PCOS patients. The purpose of this study was to elucidate its potential mechanisms and clinical significance and to determine a beneficial dietary structure and composition for PCOS patients.

## 2 Materials and methods

### 2.1 Search strategy and selection criteria

We followed the PRISMA guidelines for this systematic review and conducted a series of systematic reviews and meta-analyses in accordance with generally accepted reporting guidelines (Cochrane Handbook for Systematic Reviews of Interventions, 6.0 ed. Cochrane; 2019. Available from: http://www.training.cochrane.org/handbook. This review was registered at www.crd.york.ac.uk/PROSPERO as CRD42024579681.

### 2.2 Review question (PICOTS)

The PICOTS [Population (P), Intervention (I), Comparison (C), Outcome (O), Time (T), and Study Design (S)] criteria were established before the literature search. Our study question was, in females with PCOS (P), compared with the low-fiber, low-whole-grain, high-GI/high-GL (C), high-fiber, high-whole-grain, and low glycemic index (LGI)/low glycemic load (LGL) (I) diets in randomized clinical trials (RCTs) (S) on whether treatment can improve cardiovascular and reproductive health (O) within 6 h to 6 months (T). The intervention duration ranged from 6 h to 6 months. Although short-term studies (e.g., 6 h) were limited in number, they were included due to their relevance in assessing immediate postprandial metabolic effects, particularly for hormonal markers such as testosterone and SHBG. Sensitivity analyses excluding short-term studies confirmed the robustness of the main results.

### 2.3 Primary and secondary outcomes

Our primary outcomes focused on the effects of carbohydrate quality on sex hormones, glycolipid metabolism, and anthropometric parameters in patients with PCOS. The representative indicators include total testosterone (TT), the free androgen index (FAI), sex hormone-binding globulin (SHBG), fasting glucose, homeostatic model assessment of insulin resistance (HOMA-IR), total cholesterol (TC), low-density lipoprotein cholesterol (LDL-C), high-density lipoprotein cholesterol (HDL-C), triglycerides (TG), waist circumference (WC), and body weight. The secondary outcomes included fasting insulin, dehydroepiandrosterone sulfate (DHEAS), luteinizing hormone (LH), and follicle-stimulating hormone (FSH) levels.

### 2.4 Data sources and search strategy

Following the PRISMA statement, we used the PICOTS framework and conducted a comprehensive search across multiple electronic databases, including Ovid MEDLINE, Embase, PubMed, Web of Science, and Scopus, for articles published up to January 1, 2024. The keywords and subject headings used in the search are detailed in Supplementary material. The reference lists of the included studies were also manually reviewed to identify additional relevant trials. No language restrictions were applied, but unpublished studies were excluded because they were beyond the scope of this review. To date, no additional studies meeting the inclusion criteria have been identified.

### 2.5 Inclusion and exclusion criteria

**FIGURE 1:**
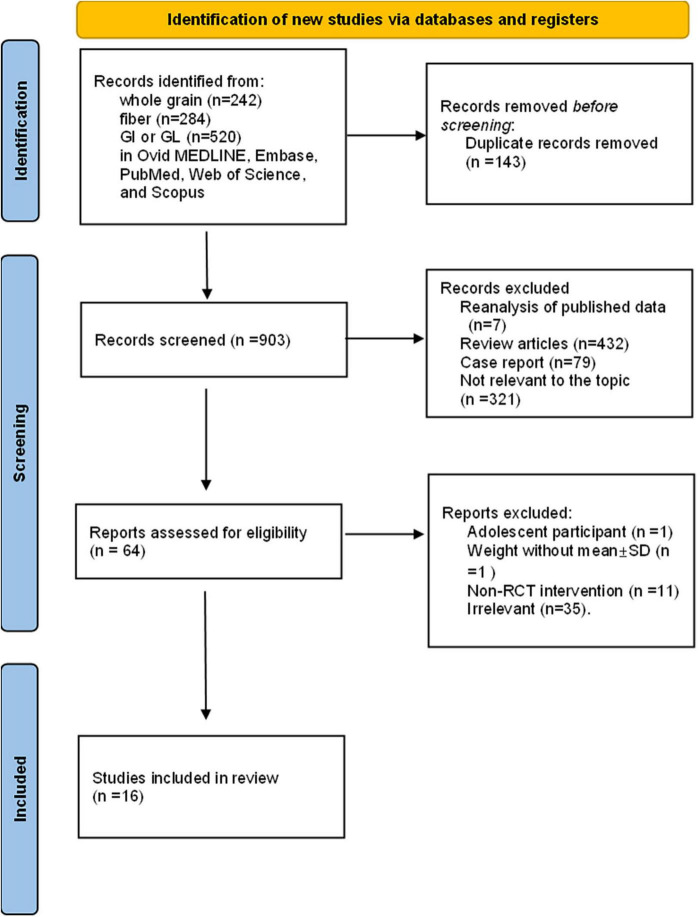
Flow diagram of the literature search.

Eligible studies were identified through screening of titles, abstracts, and full-text articles. The inclusion and selection process is illustrated in the PRISMA flow diagram (Figure 1). Studies meeting the PICOTS criteria were included. We focused on parallel or crossover RCTs involving women of reproductive age with PCOS, assessing the impact of diets rich in fiber, whole grains, and low GI/GL on sex hormones, cardiovascular outcomes, and body weight. The exclusion criteria were non-peer-reviewed publications, non-RCT studies, duplicate reports of the same trial, studies involving adolescents, pregnant women, infertile women without PCOS, non-human models, or studies with unretrievable data after the corresponding author was contacted. Two researchers (ZL and JYX) independently conducted the inclusion and exclusion process via the double-blind code assignment function in EPPI-Reviewer 4 (EPPI-Centre Software; UCL Institute of Education) and EndNote X9.2 (Thomson Reuters). Discrepancies were resolved through discussion with a third investigator (ZGH).

### 2.6 Data extraction

Data were extracted using a standardized protocol and included the following: (1) first author’s name; (2) publication year; (3) study location; (4) participant characteristics, including total sample size, number of participants completing each intervention and control group, baseline age, BMI, and PCOS diagnosis; (5) study design; (6) study duration; (7) dietary characteristics, including energy and macronutrient composition for both groups; and (8) changes in blood glucose metabolism, cardiometabolic indicators, reproductive outcomes, and body measurements between the intervention and control groups (Table 1). In cases of missing or unclear data, the corresponding author was contacted via email twice to obtain the necessary information. Data extraction was performed by one investigator (ZL) and reviewed by the remaining authors (JYX, ZGH) to minimize potential errors.

**TABLE 1:** Characteristics of the included studies.

References, country	Participants’ characteristics [mean age (y), mean BMI (kg/m^2^)]	Number of participants	PCOS definition	RCT design and duration	Intervention diet characteristics [type, composition (%, mean, or range)]	Control diet characteristics [type, composition (%, mean, or range)]	Reported outcomes of interest
Foroozanfard et al. (32), Iran	Intervention, age: 27.1, BMI, 32.3; Control, age: 25.6; BMI, 32.2; overweight or obese patients	Total completers, 60 (Intervention, 30; Control, 30)	Rotterdam	Parallel, 12 wk	LGI: standard DASH dietary pattern; E%: CHO, 52–55%; F, 30%; P, 16–18%; fiber: 18 g/d; target energy deficit, 350–700 kcal/d	HGI: Iranian traditional dietary pattern; E%: CHO, 52–55%; F, 30%; P, 16–18%; fiber: 12 g/d; target energy deficit, 350–700 kcal/d	↓Fasting insulin, ↔fasting glucose, ↓HOMA-IR, ↓weight, ↔TT, ↓FAI
Kazemi et al. (25, 42), Canada	Intervention, age: 27.0, BMI, 32.5; Control, age: 26.9, BMI, 33.3	Total completers, 61 (Intervention, 30; Control, 31)	AEPCOS 2006 (with AEPCOS 2013 criterion of ≥ 25 FNPO or OV ≥ 10 mL for PCO)	Parallel, 16 wk	LGI: low-GI pulse-based diet; E%: CHO, 52–55%; F, 30%; P, 16–18%; fiber: 33 g/d; GI:∼35–40; GL: ∼70–100	HGI: standard TLC diet; E%: 52–55%; F, 30%; P, 16–18%; fiber: 25 g/d; GI: ∼50–60; GL:∼100–110	↔Fasting insulin, ↔fasting glucose, ↔HOMA-IR, ↔TC, ↓LDL-C, ↔HDL-C, ↓TG, ↔weight, ↔WC↔TT, ↔FAI
Asemi et al. (35) and (33) Iran	Intervention, age: 22.1; BMI, 30.3; Control, age: 24.7, BMI, 28.6; overweight and obese women	Total completers, 48 (Intervention, 24; Control, 24)	Rotterdam	Parallel, 8 wk	LGI: standard DASH dietary pattern; E%: CHO, 52%; F, 30%; P, 18%; fiber: 17 g/d; target energy deficit, 350–700 kcal/d	HGI: Iranian traditional dietary pattern; E%: CHO, 52%; F, 30%; P, 18%; fiber: 12 g/d; target energy deficit: 350–700 kcal/d	↓Fasting insulin, ↔fasting glucose, ↓HOMA-IR, ↔TC, ↔LDL-C, ↔HDL-C, ↓TG, ↓weight, ↓WC
Turner-McGrievy et al. (34), USA	Intervention, age: 28.1, BMI, 42.7; Control, age: 27.4, BMI, 37.2; overweight or obese	Total completers, 18 (Intervention, 9, Control, 9);	Rotterdam	Parallel, 6 mo	LGI: vegan low-GI diet; E%: NR; fiber: NR	HGI: conventional hypocaloric diet; E%: NR; fiber: NR; target energy intake: 1200 or 1,500 kcal/d	↔Weight
Azadi-Yazdi et al. (36), Iran	Intervention, age: 32.1, BMI, 31.9; Control, age: 31.8; BMI, 30.2; Overweight and obese	Total completers, 55 (Intervention, 28;Control, 27)	Rotterdam	Parallel, 12 week	LGI: standard DASH dietary pattern; E%: CHO, 50–55%; F, 25–30%; P, 15–20%; fiber: 18 g/d; target energy deficit, 350–500 kcal/d	HGI: control diet; E%: CHO, 50–55%; F, 25–30%; P, 15–20%; fiber: 13 g/d; target energy deficit, 350–500 kcal/d	↓Weight, ↔WC, ↓TT, ↓FAI
Gower et al. (10) and Goss et al. (6), USA	Age: 31.2, BMI, 31.8	Total completers, 50 (Intervention, 27; Control, 23)	NIH	Crossover, 8 wk in 2 periods; (4-week washout period)	LGI: low-CHO diet; E%: CHO, 41%; F, 40%; P, 19%; fiber: 22–31 g/d; GI: ∼50; GL: 81–114; target energy intake, 1,800–2,500 kcal/d	HGI: standard diet; E%: CHO, 55%; F, 27%; P, 18%; fiber: 18–23 g/d; GI∼60, GL:143–192; target energy intake, 1,800–2,500 kcal/d	↓Fasting insulin, ↓fasting glucose, ↓HOMA-IR, ↓TC, ↓LDL-C, ↓HDL-C, ↔TG, ↔weight, ↓TT, ↔FAI
Atiomo et al. (37), UK	Intervention, age: 35.3, BMI, 45.1; Control, age: 36.4, BMI, 38.9	Total completers, 11 (Intervention, 6; Control, 5)	Rotterdam	Parallel, 6 mo	LGI: low-GI diet; E%: NR; fiber: NR; target energy deficit, 600 kcal/d	HGI: conventional healthy diet; E%: NR; fiber: NR; target energy deficit, 600 kcal/d	↔Fasting insulin, ↔fasting glucose, ↔TC, ↔LDL-C, ↔HDL-C, ↔TG, ↔weight, ↔WC, ↔TT
Katcher et al. (43), USA	Age: 26.9 BMI, 29.6	Total completers, 30 (Intervention, 15; Control, 15)	Self-defining^1^	Crossover, 6 h	High fiber: isocaloric low-fat, high-fiber meal (HIFIB); E%: F: 6%, CHO: 81%, fiber: 27 g	Low fiber: high-fat, Western meal (HIFAT); E%: F, 62%; CHO:24%; fiber: 1 g	↔TT, ↔FAI, ↔SHBG
Dou et al. (38), China	Intervention, age: 31, BMI, 27.87; Control, age: 31, BMI, 27.38; overweight or obese	Total completers, 60 (Intervention, 30; Control, 30)	Rotterdam	Parallel, 8 week	High fiber: target energy intake: 1 000∼1 200 kcal/d,E%: CHO, 55%∼60%,F, 25%∼30%; P 1.5∼2.0 g/kg/d, whey protein 30 g/d, fiber: 10 g/d (inulin: 83.3%; xylooligosaccharide: 16.7%)	Low fiber: target energy intake: 1 000∼1 200 kcal/d, E%: CHO, 55%∼60%, F, 25%∼30%; P 1.5∼2.0 g/kg/d, whey protein 30 g/d	↓fasting glucose, ↓HOMA-IR,↓Fasting insulin, ↑SHBG, ↔TT, ↔weight, ↔FAI
Manzoor et al. (14), Pakistan	Intervention 1, age: 25, BMI, 27.5; Intervention 2, age: 25, BMI, 27.5; Control, age: 25, BMI, 27.5	Total completers, 27 (Intervention 1, 9; Intervention 2, 9; Control, 9)	ESHRE/ASRM sponsored PCOS consensus	Parallel, 2 mo	High fiber 1: sodium alginate 3:E%: CHO, 117 g; P, 117 g; F, 52 g; 0.03 g/kg/d sodium alginate; target energy intake 1400 kcal/d High fiber 2: sodium alginate 6: E%: CHO, 117 g; P, 117 g; F, 52 g; 0.06 g/kg/d sodium alginate; target energy intake 1400 kcal/d	Low fiber: E%: CHO, 117 g; P, 117 g; F, 52 g; 0.03 g/kg/d sodium alginate; metformin 500 mg day/1; target energy intake 1400 kcal/d	↔fasting glucose, ↑HDL-C, ↓LDL-C, ↓TC, ↔weight, ↔TG
Mehrabani et al. (40), Iran	Intervention, age: 30.5, BMI, 31.9; Control, age: 28.5, BMI, 31.1	Total completers, 49 (Intervention, 23; Control, 26)	NIH	Parallel, 12 wk	LGL: low-GL hypocaloric diet; E%: CHO, 40%; F, 30%; P, 30%; fiber: NR; focus on carbohydrate intake from low- and medium-GL foods and limiting high-GL foods and refraining from high-GL (≥ 20) foods; target energy deficit, 500–1,000 kcal/d	HGL: conventional hypocaloric diet; E%: CHO, 55%; F, 30%; P, 15%; fiber: NR; target energy deficit, 500–1000 kcal/d;	↓Fasting insulin, ↔fasting glucose, ↓HOMA-IR, ↔TC, ↔LDL-C, ↔HDL-C, ↔TG, ↔weight, ↓WC, ↔TT, ↔FAI
Panico et al. (39) Italy	Age: 23.4, BMI, 28.7	Total completers, 14 (Intervention, 7; Control, 7)	Modified Rotterdam (9 FNPO 2–8 mm and DHEAS > 248 μg/dL)	Parallel, 3 mo in 2 periods; (washout period, NR)	LGL: low-GL diet; E%: CHO, 44–45%; F, 37–38%; P, 18%; fiber: 25–35 g/d; GI: 63–69; GL: 79–105; target energy intake, 1,500–1,800 kcal/d	HGL: moderately high GL diet; E%: CHO, 50–52%; F, 29–30%; P, 19–20%; fiber: 34–44 g/d; GI, 66–68; GL, 123–134; target energy intake, 1500–1800 kcal/d	↔Fasting insulin, ↔fasting glucose, ↔HOMA-IR, ↔TC, ↔TG, ↔weight, ↓TT
Hoover et al. (41) USA	Age: 31.2, BMI, 31.57	Total completers, 54 (Intervention, 27; Control, 27)	NIH	Crossover, 8 wk in 2 periods; (4-wk washout period)	LGL: low GL diet; E%: CHO, 41%; F, 40%; P, 19%, average GL was 48.27/1000 kcal	HGL: low GL diet; E%: CHO, 55%; F, 27%; P, 18%; average GL was 83.31/1,000 kcal	↔fasting glucose, ↔Fasting insulin,

^1^AEPCOS, androgen excess and polycystic ovary syndrome; CHO, carbohydrate; DASH, Dietary Approaches to Stop Hypertension; DHEAS, dehydroepiandrosterone sulfate; E, energy; F, fat; FAI, free androgen index; fiber, total dietary fiber; FNPO number, follicle per ovary; GI, glycemic index; GL, glycemic load; HDL-C, HDL cholesterol; HGI, higher glycemic index diet; HGL, higher glycemic load; LDL-C, LDL cholesterol; LGI, lower glycemic index; LGL, lower glycemic load; NR, not reported; OV, ovarian volume; P, protein; PCO, polycystic ovaries; PCOS, polycystic ovary syndrome; RCT, randomized controlled trial; TC, total cholesterol; TG, triglyceride; TLC, therapeutic lifestyle changes; TT, total testosterone; WC, waist circumference.; wk, week; mo, month; ↓Denotes decreases in evaluated outcome measures in LGI diets compared with HGI diets, and ↔ denotes comparable effects of LGI and HGI diets. Self-defining 1: [1] had a history of chronic anovulation, determined by intermenstrual periods of ≥ 45 days or ≤ 8 menstrual cycles/year, and [2] had hyperandrogenism, determined by total T > 50 ng/dL or a free androgen index (FAI) > 1.5. Hyperprolactinemia was excluded by a PRL level ≤ 25 ng/mL, non-classic adrenal 21-hydroxylase deficiency was excluded by a 17a-hydroxyprogesterone (17-OHP level < 2ng/mL), and thyroid disorders were excluded by a TSH level ≥ 0.2 mIU/mL or ≤ 5.5 mIU/m.

### 2.7 Assessment of risk of bias

The Cochrane risk-of-bias assessment tool was employed to evaluate the risk of bias in the RCTs across seven domains: (1) sequence generation, (2) allocation concealment, (3) blinding of participants and personnel, (4) blinding of outcome assessors, (5) incomplete outcome data (attrition bias), (6) selective reporting, and (7) other biases. Each domain was rated as “high,” “low,” or “unclear” risk of bias. The quality assessment for each included study was conducted independently by two investigators (ZL and JYX), with any discrepancies resolved through consensus or consultation with a third investigator (ZGH).

### 2.8 Methods of analysis

Statistical analysis was conducted using Review Manager 5.3 and STATA V.18. Effect sizes in the experimental and control groups were expressed as Standardized Mean Difference (SMD) with 95% Cis. Pooled effects were estimated using mean differences and standard deviations (SDs) if reported in three or more trials. A fixed-effects model was applied when heterogeneity was < 50%, while a random-effects model was used for heterogeneity ≥ 50%. When the net changes were not directly reported, the mean changes were calculated by subtracting the baseline values from the postintervention results.

Heterogeneity was assessed using the chi-square test, and I^2^ was reported to indicate heterogeneity, with < 50% indicating low heterogeneity and ≥ 50% indicating high heterogeneity. Subgroup and sensitivity analyses were performed to explore sources of heterogeneity. Subgroup analyses examined the impact of (1) participants’ age (≤ 30 years or > 30 years), (2) energy restriction in the intervention and control groups (yes or no), and (3) duration of the intervention (< 16 or ≥ 16 weeks). Each subgroup included two or more studies. The sensitivity analysis involved excluding one study at a time to assess its impact on the overall effect size and heterogeneity, determining if any single trial had an undue influence.

Publication bias was evaluated using Begg’s rank correlation test and Egger’s regression asymmetry test. A *p*-value of ≤ 0.05 was considered statistically significant.

## 3 Results

### 3.1 Data selection and study characteristics

A total of 1,046 records were retrieved from electronic databases, including 242 related to whole grain, 284 to fiber, and 520 to GI or GL. After removal of duplicates, 903 records remained. Based on title and abstract screening, 64 full-text articles were reviewed. Articles were excluded for the following reasons: reanalysis of published data (*n* = 7), review articles (*n* = 432), case reports (*n* = 79), and studies not relevant to the topic (*n* = 321). After full-text assessment, additional exclusions were made: studies involving adolescent participants (*n* = 1), studies lacking weight data with mean ± SD (*n* = 1), non-RCT interventions (*n* = 11), and irrelevant content (*n* = 35). Ultimately, 16 articles met the eligibility criteria and were included in this meta-analysis. These studies, published between 2009 and 2023, were conducted in the United States, Canada, the United Kingdom, Iran, Italy, China, and Pakistan. In the intervention group and the control group, the mean age and BMI (kg/m^2^) of the females were 28.04 years and 31.93 kg/m^2^, respectively. Most of the included RCTs used the Rotterdam criteria to define PCOS (32–39), other NIH standards (6, 10, 40, 41) and AEPCOS standards (14, 25, 43, 42). Among all 13 included RCTs, 10 studies had parallel designs, and three studies used crossover designs. The intervention time range was 6 h–6 months. The dietary intervention had no energy restrictions or energy restrictions. The results of blood glucose metabolism, blood lipid metabolism, reproductive indicators, and anthropometric measurements included fasting glucose, fasting insulin, HOMA-IR, TC, LDL-C, HDL-C, TG, TT, FAI, SHBG, DHEAS, LH, FSH, body weight, and WC.

### 3.2 Quality assessment

Using the Cochrane risk of bias tool, we evaluated the included RCTs as having moderate quality. Among these studies, 87% had a low risk of bias for random sequencing, 30% for allocation concealment, 60% for blinding of participants and personnel, 50% for blinding of outcome assessment, 65% for incomplete outcome data, 100% for selective reporting and 100% for other bias (Supplementary Table 1).

### 3.3 Meta-analyses

#### 3.3.1 Effect of carbohydrate quality on glucose metabolism in women with PCOS

##### 3.3.1.1 Fasting glucose

**FIGURE 2:**
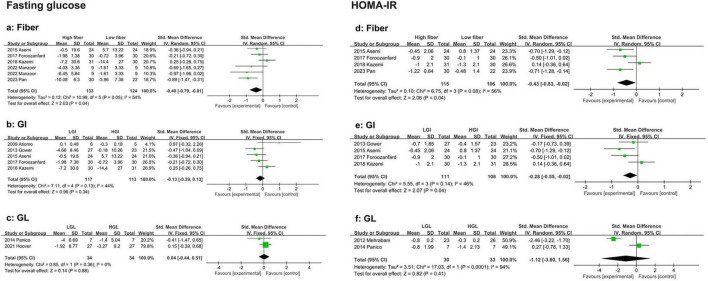
Forest plots showing the effects of high-fiber, LGI, and LGL dietary patterns on glucose metabolism in randomized controlled trials in PCOS women. **(a)** High-fiber dietary pattern on fasting glucose; **(b)** LGI dietary pattern on fasting glucose; **(c)** LGL dietary pattern on fasting glucose; **(d)** High-fiber dietary pattern on HOMA-IR; **(e)** LGI dietary pattern on HOMA-IR; **(f)** LGL dietary pattern on HOMA-IR.

A pooled analysis of six eligible studies revealed that a high-fiber diet significantly reduced fasting blood glucose compared with a low fiber diet (SMD: −0.40, 95% CI: −0.79 to −0.01, *P* = 0.04, I^2^ = 54%) (Figure 2a). Sensitivity analysis indicated that removing the study by Asemi et al. (33) (SMD: −0.55, 95% CI: −0.86 to −0.24, *P* < 0.01, I^2^ = 10.9%) substantially reduced heterogeneity. No evidence of publication bias was detected (Begg’s test *P* = 0.06, Egger’s test *P* = 0.192) (Supplementary Data).

Five studies compared the reduction in fasting blood glucose between LGI and HGI diet interventions in PCOS patients. Meta-analysis revealed a reduction in fasting blood glucose in the LGI group (Figure 2b) although the difference was not statistically significant in fasting blood glucose (SMD: −0.34, 95% CI: −0.65 to −0.02, *P* = 0.03, I^2^ = 0%) than a longer duration (≥ 16 weeks; SMD: 0.34, 95% CI: −0.13 to 0.81, *P* = 0.15, I^2^ = 4%) (Supplementary Figure 1a). No publication bias was found (Begg’s test *P* = 1, Egger’s test *P* = 0.355) (Supplementary Data).

Only two studies have compared the effects of HGL and LGL diets on fasting blood glucose in PCOS patients. The pooled results revealed no significant difference between the two groups (SMD: 0.04, 95% CI: −0.44 to 0.51, *P* = 0.88, I^2^ = 0%) (Figure 2c).

##### 3.3.1.2 Homeostatic model assessment of insulin resistance (HOMA-IR)

This meta-analysis included four eligible studies. Compared with a low-fiber diet, a high-fiber diet significantly reduced HOMA-IR (SMD: −0.43, 95% CI: −0.83 to −0.02, *P* = 0.04, I^2^ = 56%) (Figure 2d). After excluding the study by Kazemi et al. (42), heterogeneity was eliminated (SMD: −0.64, 95% CI: −0.95 to −0.32, *P* < 0.01, I^2^ = 0%). No evidence of publication bias was found (Begg’s test *P* = 0.308, Egger’s test *P* = 0.188) (Supplementary Data).

Five studies demonstrated that an LGI diet significantly reduced HOMA-IR in women with PCOS compared with an HGI diet (SMD: −0.28, 95% CI: −0.55 to −0.02, *P* = 0.04, I^2^ = 46%) (Figure 2e). In the energy-restricted subgroup, the low-GI diet further reduced HOMA-IR (SMD: −0.59, 95% CI: −0.97 to −0.20, *P* < 0.01, I^2^ = 0%) (Supplementary Figure 1b). No publication bias was detected (Begg’s test *P* = 0.308, Egger’s test *P* = 0.376) (Supplementary Data).

Only two studies have examined the impact of GL on HOMA-IR in PCOS patients. The meta-analysis indicated no significant difference between the LGL and HGL diets in reducing HOMA-IR (SMD: −1.12, 95% CI: −3.80 to 1.56, P = 0.41, I^2^ = 94%) (Figure 2f).

The effect of carbohydrate quality on fasting insulin in women with PCOS was similar to the findings for HOMA-IR and is reported in Supplementary Figure 2.

#### 3.3.2 Effect of carbohydrate quality on lipid metabolism in women with PCOS

##### 3.3.2.1 Low-density lipoprotein cholesterol

**FIGURE 3:**
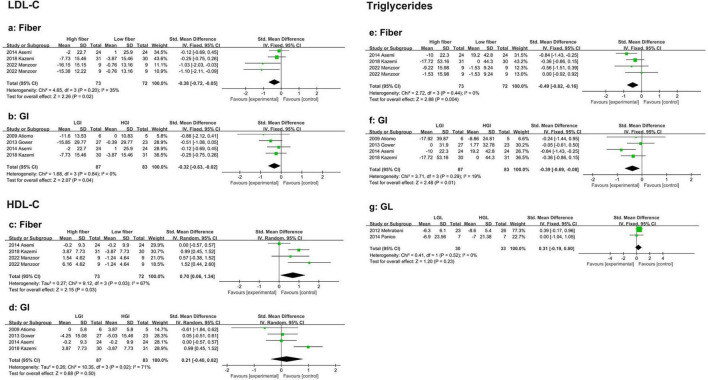
Forest plot of the effects of a high-fiber, LGI, and LGL dietary patterns on lipid metabolism in randomized controlled trials in PCOS women. **(a)** High-fiber dietary pattern on LDL-C; **(b)** LGI dietary pattern on LDL-C; **(c)** High-fiber dietary pattern on HDL-C; **(d)** LGI die-tary pattern on HDL-C; **(e)** High-fiber dietary pattern on TG; **(f)** LGI dietary pattern on TG; **(g)** LGL dietary pattern on TG.

This meta-analysis included four studies comparing the effects of low-fiber and high-fiber diets on LDL-C levels in PCOS patients. Compared with a low-fiber diet, a high-fiber diet significantly reduced LDL cholesterol (SMD: −0.38, 95% CI: −0.72 to −0.05, *P* = 0.02, I^2^ = 35%) (Figure 3a). No evidence of publication bias was detected (Begg’s test *P* = 0.308, Egger’s test *P* = 0.040) (Supplementary Data).

Similarly, four studies were included in the analysis that compared the effects of LGI and high-GI diets on LDL-C levels in PCOS patients. The results showed that an LGI diet was more effective at reducing LDL-C (SMD: −0.32, 95% CI: −0.63 to −0.02, *P* = 0.04, I^2^ = 0%) (Figure 3b). No publication bias was observed (Begg’s test *P* = 0.734, Egger’s test *P* = 0.287) (Supplementary Data).

##### 3.3.2.2 High-density lipoprotein cholesterol

Four eligible studies reported a significant increase in HDL-C with high-fiber diets (SMD: 0.70, 95% CI: 0.06 to 1.34, *P* = 0.03, I^2^ = 67%) (Figure 3c). Sensitivity analysis revealed that the studies by Asemi et al. (35) were excluded. Significantly reduced heterogeneity (SMD: 1.01, 95% CI: 0.59 to 1.44, *P* < 0.01, I^2^ = 0%). No evidence of publication bias was found (Begg’s test *P* = 0.734, Egger’s test *P* = 0.603) (Supplementary Data).

Four studies evaluated the effects of LGI versus HGI diets on HDL-C in PCOS women. The pooled results indicated no significant difference between the two diets in their impact on HDL cholesterol levels (SMD: 0.21, 95% CI: −0.40 to 0.82, *P* = 0.50, I^2^ = 71%) (Figure 3d). Sensitivity analysis revealed that, after excluding the study by Kazemi et al. (42), heterogeneity was significantly reduced, although the effect remained statistically insignificant (SMD: −0.04, 95% CI: −0.42 to 0.34, *P* = 0.84, I^2^ = 0%). Subgroup analysis revealed that in participants aged > 30 years (SMD: −0.06, 95% CI: −0.57 to 0.44, *P* = 0.81, I^2^ = 0%), those in the energy-restricted group (SMD: −0.11, 95% CI: −0.62 to 0.41, *P* = 0.68, I^2^ = 0%), and those with a study duration of < 16 weeks (SMD: 0.03, 95% CI: −0.37 to 0.42, *P* = 0.90, I^2^ = 0%), heterogeneity was significantly reduced, indicating that age, energy restriction, and study duration contributed to the heterogeneity. However, the effect estimates did not differ between subgroups (Supplementary Figures 3a–c). No evidence of publication bias was found (Begg’s test *P* = 0.089, Egger’s test *P* = 0.445) (Supplementary Data).

##### 3.3.2.3 Triglycerides

Four eligible studies demonstrated that the reduction in triglyceride (TG) levels was significantly greater in the high-fiber diet group than in the low-fiber diet group (SMD: −0.49, 95% CI: −0.82 to −0.16, *P* < 0.01, I^2^ = 0%) (Figure 3e). No evidence of publication bias was found (Begg’s test *P* = 1.00, Egger’s test *P* = 0.754) (Supplementary Data).

Similarly, four studies indicated that the LGI diet group experienced a significantly greater reduction in TG levels than the HGI diet group did (SMD: −0.39, 95% CI: −0.69 to −0.08, *P* = 0.01, I^2^ = 19%) (Figure 3f). No publication bias was detected (Begg’s test *P* = 1.00, Egger’s test *P* = 0.993) (Supplementary Data).

Two studies reported no significant difference in TG reduction between HGL and LGL diets (SMD: 0.31, 95% CI: −0.19 to 0.80, *P* = 0.23, I^2^ = 0%) (Figure 3g).

##### 3.3.2.4 Total cholesterol

The effect of a high fiber and LGI diet on TC in women with PCOS was generally consistent with the results observed for TG, as detailed in Supplementary Figure 4. However, two eligible studies evaluated the impact of LGL versus HGL diets on TC levels in PCOS patients, with the LGL diet significantly reducing TC (SMD: −0.63, 95% CI: −1.14 to −0.12, *P* = 0.02, I^2^ = 9%)

#### 3.3.3 Effects of carbohydrate quality on sex hormones in women with PCOS

##### 3.3.3.1 Total testosterone

**FIGURE 4:**
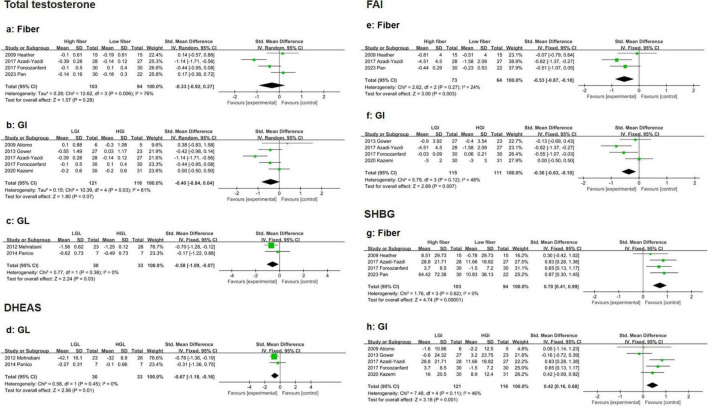
Forest plot of the effects of a high-fiber, LGI, and LGL dietary patterns on sex hormones in randomized controlled trials in PCOS women. **(a)** High-fiber dietary pattern on TT; **(b)** LGI dietary pattern on TT; **(c)** LGL dietary pattern on TT; **(d)** LGL dietary pattern on DHEAS; **(e)** High-fiber dietary pattern on FAI; **(f)** LGI dietary pattern on FAI; **(g)** High-fiber dietary pattern on SHBG; **(h)** LGI dietary pattern on SHBG.

Four eligible studies indicated that high-fiber and low-fiber dietary interventions had no significant effect on total testosterone levels in PCOS patients (SMD: −0.33, 95% CI: −0.92 to 0.27, *P* = 0.28, I^2^ = 76%) (Figure 4a). The sensitivity analysis excluded the study by Azadi-Yazdi et al. (36). Significantly reduced heterogeneity (SMD: −0.07, 95% CI: −0.49 to 0.35, *P* = 0.734, I^2^ = 35.8%). Subgroup analysis on the basis of age revealed no significant difference in the reduction in TT between participants aged > 30 years (SMD: −0.48, 95% CI: −1.76 to 0.80, *P* = 0.46, I^2^ = 90%) and those aged ≤ 30 years (SMD: −0.20, 95% CI: −0.76 to 0.35, *P* = 0.48, I^2^ = 40%) (Supplementary Table 2). No evidence of publication bias was detected (Beg’s test *P* = 1.000, Egger’s test *P* = 0.715) (Supplementary Data).

Five eligible studies suggested that the LGI diet had a greater effect on reducing total testosterone levels than did the HGI diet in PCOS patients, although the results did not reach statistical significance (SMD: −0.40, 95% CI: −0.84 to 0.04, *P* = 0.07, I^2^ = 61%) (Figure 4b). Sensitivity analysis revealed that removing Azadi-Yazdi et al. (36). Significantly reduced heterogeneity (SMD: −0.24, 95% CI: −0.53 to 0.06, *P* = 0.117, I^2^ = 1.0%). Subgroup analysis based on energy restriction revealed that the LGI diet was more effective at reducing total testosterone when the intervention duration was shorter (<16 weeks) (SMD: −0.66, 95% CI: −1.11 to −0.20, *P* < 0.01, I^2^ = 51%) (Supplementary Figure 1c). No publication bias was observed (Begg’s test *P* = 0.806, Egger’s test *P* = 0.703) (Supplementary Data).

Compared with the HGL diet, the LGL diet resulted in a significantly greater reduction in total testosterone in PCOS patients (SMD: −0.58, 95% CI: −1.09 to −0.07, *P* = 0.03, I^2^ = 0%) (Figure 4c).

##### 3.3.3.2 Dehydroepiandrosterone sulfate (DHEAS)

Two eligible studies revealed that the reduction in DHEAS levels was significantly greater in the LGL group than in the HGL group (SMD: −0.67, 95% CI: −1.18 to −0.16, *P* = 0.01, I^2^ = 0%) (Figure 4d).

##### 3.3.3.3 Free androgen index

Four eligible studies demonstrated that the reduction in FAI was significantly greater in the high-fiber diet group than in the low-fiber diet group (SMD: −0.53, 95% CI: −0.87 to −0.18, *P* < 0.01, I^2^ = 24%) (Figure 4e). No evidence of publication bias was detected (Begg’s test *P* = 0.308, Egger’s test *P* = 0.217) (Supplementary Data).

Similarly, four studies reported that the reduction in FAI was more pronounced in the LGI group than in the HGI group (SMD: −0.36, 95% CI: −0.63 to −0.10, *P* < 0.01, I^2^ = 48%) (Figure 4f). Subgroup analysis indicated that in the energy-restricted subgroup, the improvement in FAI was even greater in the LGI subgroup (SMD: −0.68, 95% CI: −1.05 to −0.30, *P* < 0.01, I^2^ = 0%) (Supplementary Figure 1d). No publication bias was observed (Begg’s test *P* = 0.734, Egger’s test *P* = 0.516) (Supplementary Data).

##### 3.3.3.4 Sex hormone-binding globulin

Compared with a low-fiber diet, a high-fiber diet significantly increased SHBG levels in four eligible studies (SMD: 0.70, 95% CI: 0.41 to 0.99, *P* < 0.01, I^2^ = 0%) (Figure 4g). No evidence of publication bias was detected (Begg’s test *P* = 1.000, Egger’s test *P* = 0.303) (Supplementary Data). Five eligible studies demonstrated that the LGI diet significantly improved SHBG levels compared with the HGI diet (SMD: 0.42, 95% CI: 0.16 to 0.68, *P* < 0.01, I^2^ = 46%) (Figure 4h). Subgroup analysis revealed that the improvement in SHBG was more pronounced in younger participants (≤ 30 years, SMD: 0.53, 95% CI: 0.17 to 0.89, *P* < 0.01, I^2^ = 0%) and in those with energy restrictions (SMD: 0.67, 95% CI: 0.31 to 1.03, *P* < 0.01, I^2^ = 0%) (Supplementary Figure 1e). The LGI diet consistently showed greater benefits than the HGI diet across these subgroups (Supplementary Table 2). No publication bias was observed (Begg’s test *P* = 0.806, Egger’s test *P* = 0.671) (Supplementary Data).

Compared with an HGI diet, an LGI diet does not significantly impact LH or FSH levels; these results are presented in Supplementary Figure 5.

#### 3.3.4 Effects of carbohydrate quality on the anthropometrics of women with PCOS

##### 3.3.4.1 Weight

**FIGURE 5:**
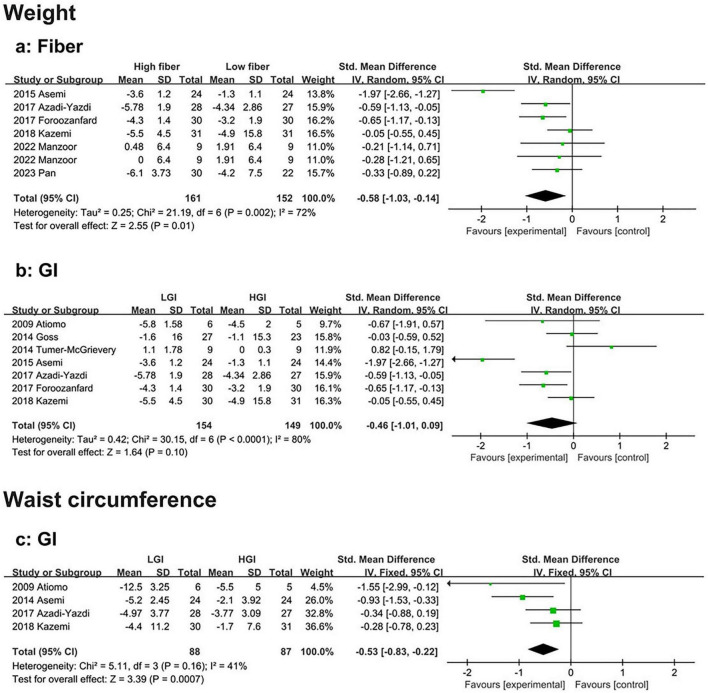
Forest plot of the effects of a high-fiber, LGI, and LGL dietary patterns on anthropometrics in randomized controlled trials in PCOS women. **(a)** High-fiber dietary pattern on weight; **(b)** LGI dietary pattern on weight; **(c)** LGI dietary pattern on waist circumference.

Seven eligible studies demonstrated that body weight loss was significantly greater in the high-fiber diet group than in the low-fiber diet group (SMD: −0.58, 95% CI: −1.03 to −0.14, *P* = 0.01, I^2^ = 72%) (Figure 5a). Sensitivity analysis revealed that heterogeneity was significantly reduced after excluding the study by Asemi et al. (33). (SMD: −0.38, 95% CI: −0.63 to −0.14, *P* < 0.01, I^2^ = 0%). Subgroup analysis revealed that the weight loss effect was more pronounced in participants aged > 30 years (SMD: −0.46, 95% CI: −0.85 to −0.08, *P* = 0.02, I^2^ = 0%). No publication bias was detected (Supplementary Figure 6a). No evidence of publication bias was detected (Begg’s test *P* = 0.764, Egger’s test *P* = 0.687) (Supplementary Data).

Seven eligible studies also revealed no significant difference in body weight loss between the HGI and LGI diet groups (SMD: −0.46, 95% CI: −1.01 to 0.09, *P* = 0.10, I^2^ = 80%) (Figure 5b). However, the sensitivity analysis excluded the study by Tumer-McGrievery et al. (34). There was greater weight loss in the LGI diet group than in the HGI group, with a significant difference between the two groups (SMD: −0.65, 95% CI: −1.20 to −0.10, *P* = 0.021, I^2^ = 79.1%). Additionally, Asemi et al. (33). slightly reduced heterogeneity (SMD: −0.24, 95% CI: −0.62 to 0.15, *P* = 0.231, I^2^ = 53.4%). Subgroup analysis revealed that, compared with the HGI diet, the LGI diet had a greater effect on weight loss when the study duration was shorter (< 16 weeks) (SMD: −0.78, 95% CI: −1.49 to −0.07, *P* = 0.03, I^2^ = 84%) (Supplementary Figure 6b). No evidence of publication bias was found (Begg’s test *P* = 1.000, Egger’s test *P* = 0.971) (Supplementary Data).

##### 3.3.4.2 Waist circumference

Compared to the HGI diet, four studies showed that the LGI diet resulted in a significantly greater reduction in waist circumference (SMD: −0.53, 95% CI: −0.83 to −0.22, *P* < 0.01, I^2^ = 41%) (Figure 5c). No evidence of publication bias was detected (Begg’s test *P* = 0.089, Egger’s test *P* = 0.198) (Supplementary Data).

## 4 Discussion

### 4.1 Main findings

In this systematic review and meta-analysis, we assessed the effects of carbohydrate quality on metabolic and reproductive abnormalities in women with PCOS. High dietary fiber and LGI significantly reduced fasting glucose and insulin resistance. Neither the LGI nor the LGL significantly impacted blood glucose, although the LGI diet had short-term benefits (< 16 weeks). For lipid metabolism, both high fiber intake and the LGI diet significantly reduced triglycerides and LDL, with fiber also increasing HDL. The LGL diet had limited effects on triglycerides, and data on HDL and LDL were insufficient. With respect to sex hormone regulation, high-fiber and LGI diets increased SHBG and reduced FAI. The LGI diet improved total testosterone only in the short term. Although the LGL diet reduced total testosterone and DHEAS levels, further research is needed because of the small sample size. High fiber was more effective for weight loss in women over 30 years of age. While the LGI diet did not significantly impact overall weight, it did help reduce waist circumference. In conclusion, high fiber provides broad benefits for glucose and lipid metabolism, FAI, and weight management, particularly in improving overall blood glucose and lipid profiles. The LGI diet, especially the LGL, increased androgen levels. However, the limitations of LGI/GL diets in terms of glycemic control should be noted. Finally, potential biases, imprecision, and heterogeneity among studies warrant cautious interpretation of some results. We observed moderate to high heterogeneity in the effects of dietary fiber on outcomes such as fasting glucose, HOMA-IR, HDL-C, TT, and weight. GI interventions also showed significant heterogeneity, particularly in outcomes like HDL-C, TT, and weight. Similarly, GL interventions exhibited high heterogeneity, especially in HOMA-IR.

### 4.2 Effects on glucose metabolism

In women with PCOS, insulin resistance is a key pathological feature, with an incidence rate of 75% in lean women and up to 95% in obese women; this not only reduces insulin sensitivity but also exacerbates metabolic abnormalities such as obesity and abnormal blood glucose levels (44). This study revealed that, compared with low intake, higher dietary fiber intake significantly reduces fasting blood glucose and fasting insulin levels and improves insulin resistance. However, the results for fasting blood glucose and HOMA-IR showed moderate heterogeneity. Although sensitivity analysis reduced this heterogeneity, further high-quality RCTs are needed.

Notably, GI and GL are indicators of the blood glucose response to food, and a low-GI/GL diet has been shown to improve overall blood glucose fluctuations and insulin resistance (45). However, in our study, compared with the HGI diet, the LGI diet reduced fasting insulin and improved insulin resistance but had a limited effect on fasting blood glucose. A previous meta-analysis revealed that a low-GI dietary pattern can improve postprandial blood glucose control and hyperinsulinemia, but its effect on fasting blood glucose is limited (46). Another study revealed that in healthy individuals, a low-GI diet did not significantly reduce fasting blood glucose levels, although it had positive effects on weight control and other metabolic indicators (47, 48). However, owing to heterogeneity, subgroup analysis of the GI diet after reducing heterogeneity revealed that the differences were mainly due to intervention duration. In the low-GI group (< 16 weeks) with a shorter intervention time, fasting blood glucose levels decreased significantly. This may be because, in the early stages of dietary intervention, the body responds more strongly to significant dietary changes, leading to rapid improvement in insulin sensitivity and a short-term reduction in fasting blood glucose levels (49). In contrast, the HGL diet did not significantly improve blood glucose or insulin levels. However, given the limited sample size and the high heterogeneity (94%) of the results for insulin resistance, the reliability of this conclusion is low.

In conclusion, with respect to dietary patterns that target glucose metabolism in women with PCOS, higher dietary fiber intake appears to improve glucose metabolism more comprehensively. Both increased dietary fiber intake and the LGI diet are effective dietary patterns for alleviating insulin resistance. However, further randomized controlled trials (RCTs) are needed to determine whether the LGL dietary pattern can improve insulin resistance in PCOS patients.

### 4.3 Effects on lipid metabolism

Women with PCOS generally have an unfavorable lipid profile, with lower HDL-C and higher TG and LDL-C levels, regardless of BMI (50, 51). This study revealed that a high-fiber diet significantly reduced TG and LDL levels, which is consistent with previous findings. Dietary fiber may lower LDL-C by reducing bile acid reabsorption and increasing stool volume, thereby promoting the excretion of cholesterol and its metabolites through intestinal peristalsis. Additionally, improving insulin resistance may indirectly enhance lipid metabolism in women with PCOS (52, 53) Although high dietary fiber intake appears to improve HDL levels, it is important to note the high heterogeneity (67%), which requires further confirmation through RCT studies. Compared with the HGI diet, the LGI diet improved TG and LDL but did not affect HDL-C. Further subgroup analysis, even after reducing heterogeneity, did not alter this conclusion. This may be due to the LGI diet reducing insulin secretion by stabilizing blood glucose fluctuations, which can improve LDL levels, while its effect on HDL is limited (54). Notably, the LGL diet only reduced TC and did not significantly improve TG levels. Given the limited number of studies on the GL diet (2 studies) and the absence of subgroup analyses, further research on higher-quality GL diets is needed.

In conclusion, in women with PCOS, higher dietary fiber intake and LGI diets effectively reduce LDL-C and TG levels. Despite the positive effects on HDL cholesterol, the high heterogeneity calls for further validation through high-quality RCTs.

### 4.4 Effects on sex hormones

Hyperandrogenism is a core feature of PCOS, with studies showing that approximately 70–80% of PCOS patients exhibit varying degrees of hyperandrogenism. Hyperandrogenism is a key biochemical marker for both diagnosing PCOS and assessing treatment efficacy (55). Even in the absence of abnormal glycolipid metabolism, hyperandrogenism is closely associated with a high prevalence of cardiometabolic risk factors (56), suggesting that hyperandrogenism independently affects the metabolism and overall health of women with PCOS.

In our study, no significant difference in total testosterone was observed between the high dietary fiber or LGI diet groups and the control group, but there was high heterogeneity between the two groups (76 and 61%, respectively). After further subgroup analysis reduced this heterogeneity, the difference in GI diet intervention timing partially explained it. The short-term LGI dietary pattern improved total testosterone levels, but this result still showed moderate heterogeneity (51%). The LGL diet significantly reduced total testosterone levels; however, owing to the small number of included studies, the credibility of this conclusion remains uncertain despite the low heterogeneity.

SHBG binds to testosterone, reducing its androgenic effects, while unbound free testosterone has greater biological activity (57). Therefore, the free androgen index (FAI) is generally considered a more sensitive indicator for assessing androgen levels. Our study revealed that a high-fiber and LGI diet significantly reduced FAI and increased SHBG levels. Given the heterogeneity associated with the LGI diet, further subgroup analysis indicated that energy restriction could explain the heterogeneity, with a significant improvement in FAI observed in the energy-restricted group. Subgroup analysis of SHBG revealed that both energy restriction and age differences contributed to the heterogeneity, with the younger age group (≤ 30 years) and the energy-restricted group showing significant improvements in SHBG levels. This finding aligns with previous research, indicating that a high-fiber and low-GI diet may improve FAI by increasing SHBG synthesis in the liver (58). Additionally, improvements in insulin resistance and hyperinsulinemia may inhibit androgen synthesis by reducing the expression of androgenic enzymes in theca cells (59, 54). Interestingly, studies on GL diets, despite lacking FAI and SHBG data, revealed that the LGL diet reduced both total testosterone and dehydroepiandrosterone sulfate (DHEAS), suggesting a potential positive effect on adrenal androgens.

In conclusion, these studies demonstrate that carbohydrate quality has a significant effect on reducing hyperandrogenism in women with PCOS. Both the LGL and short-term LGI diets were beneficial for lowering total testosterone, whereas higher dietary fiber intake and LGI diets improved FAI. However, further high-quality RCTs are necessary to confirm these findings.

### 4.5 Effects on anthropometrics

It has been reported that 50–80% of PCOS women are overweight or obese, and obesity has been shown to exacerbate the metabolic and reproductive disorders associated with PCOS (50, 60–62). Current studies have shown that weight loss can significantly improve PCOS symptoms, including insulin resistance, ovulation, and androgen levels (63). In our study, women with PCOS who followed a high-fiber dietary pattern had significantly lower body weights than those with PCOS who followed a low-fiber diet, although the degree of heterogeneity was high (72%). Subgroup analysis suggested that age differences, particularly in women over 30, could explain this heterogeneity. Although the LGI diet did not significantly reduce body weight compared with the HGI diet, it had a significant effect on reducing waist circumference in women with PCOS. When a carbohydrate diet is selected for women with PCOS, a higher intake of dietary fiber may enhance weight management, particularly in women with PCOS of older age. The LGI diet significantly improved waist circumference in women with PCOS, but its effect on body weight requires further verification.

### 4.6 Limitations

Our study has certain limitations. First, the limited number of studies and small sample sizes are primary constraints. Notably, only three publications have examined the effect of the LGL diet on PCOS, which reduces the reliability of the conclusions. Second, owing to ethical and practical considerations, the study could not ensure complete blinding, which introduces potential bias into the results (56). Third, while it is reasonable to investigate the effects of specific food components on the metabolism of PCOS patients independently, this can be challenging in practice. For example, low-GI diets often overlap with high-fiber diets, making it difficult to distinguish the metabolic effects of dietary fiber from those of the GI itself. Fourth, our study primarily includes data from North America, Europe, and Asia, which may limit its generalizability to populations in other regions such as South America, Africa, and Oceania. Future research should aim to include more diverse geographic regions to enhance the applicability of findings. Finally, there is no standardized definition of LGI/LGL or high-fiber diets in the literature. Variations in measurement methods and differing diagnostic criteria for PCOS contribute to study heterogeneity. Notably, GI variability is significant both between and within individuals, with considerable differences across food categories and geographic locations. Even minor changes in food variety, growing conditions, cooking methods, and chewing can affect the GI tract and, consequently, the GL (64). Thus, the use of the GI/GL as a measure of carbohydrate quality in real life has certain limitations. Additionally, the potential benefits of improving carbohydrate quality in addressing critical complications in women with PCOS, such as ovulation cycle irregularities, infertility, and cardiovascular disease risk, have not been investigated in this study. Although existing evidence suggests the importance of diet in managing PCOS, our meta-analysis did not include sufficient data on these specific outcomes. Therefore, further research is needed to explore how improving carbohydrate quality can impact these critical health issues, particularly in terms of reproductive health and cardiovascular risk in women with PCOS.

### 4.7 Advantages

While the importance of carbohydrate quality in the dietary management of PCOS is undeniable, previous studies have focused predominantly on its effects on the incidence of cardiovascular diseases and all-cause mortality (2, 28). To date, no studies have conducted a cross-sectional comparison of the different carbohydrate quality components in PCOS patients. This paper is the first to use a meta-analysis to systematically evaluate the specific effects of carbohydrate quality on glucose and lipid metabolism, sex hormones, and anthropometrics in women with PCOS. Second, different sensitivities and subgroup analyses were performed for the GI diet, which improved the accuracy of the results and provided relevant results. GI only evaluates the potential effect of a single food on blood glucose, whereas GL takes into account the serving amount, which can more comprehensively evaluate the actual effect of the entire diet on blood glucose. Therefore, although few dietary interventions have been reported for GL, these interventions have been summarized, and their ability to improve androgen levels has been proposed. These findings provide solid data support and a theoretical basis for the development of more accurate and scientific dietary intervention strategies.

### 4.8 Wider implications

These findings support the use of high dietary fiber, LGI/LGL, and whole-grain diet as a part of the dietary management of PCOS; however, care should be exercised in the promotion and application of some of the results, owing to the differences in effects on different metabolic components, which should be combined with the individual needs and health goals of the patient being comprehensively considered. Currently, the definitions of GI, GL and a high-fiber diet are not uniform, the measurement methods for each indicator are different, and the diagnostic criteria for PCOS are also different, which increases the heterogeneity of studies. In particular, GI and GL have important value in research. Owing to interindividual and inter food variability, the limitations of their use as a measure of carbohydrate quality in real life have constrained their promotion. Therefore, future research needs to achieve greater standardization in these areas to ensure the comparability and applicability of the study results. In addition, future studies should continue to investigate the specific mechanisms and effects of different indicators of carbohydrate quality on PCOS, such as the mechanism of action of the gut flora and metabolites in carbohydrate intervention, which will help establish more standardized definitions and individualized outcomes. Specific indicators of PCOS are used to guide dietary management in the clinical practice of PCOS. In the end, more high-quality RCTs are needed to confirm whether carbohydrate quality can further improve the ovulation cycle, infertility and cardiovascular disease risk in women with PCOS.

## Data Availability

The original contributions presented in the study are included in the article/Supplementary material, further inquiries can be directed to the corresponding author.
