# Supplementary material for: Optimizing carbohydrate quality: a path to better health for women with PCOS
Source: Front Nutr. 2025 Jun 18;12:1578459. doi: 10.3389/fnut.2025.1578459 (PMC12213572; doi:10.3389/fnut.2025.1578459)
Supplement: Supplementary file 1 [file Table_1.docx]

Supplementary Material

1.Supplementary Tables

2.Supplementary Figures

3.Publication Bias: Begg's and Egger's tests

4.Search strategy

**Supplementary Table 1 . Risk of bias assessment in each study**

|  | | | | | | | |
| --- | --- | --- | --- | --- | --- | --- | --- |
| Author, year (reference) | Allocation sequence generation | Allocation concealment | Blinding participants and personnel | Blinding outcome assessment | Incomplete outcome reporting | Selective outcome reporting | Other sources of bias |
| Asemi *et al.*, 2014 and 2015 | Low | Unclear | Low | Low | Low | Low | Low |
| Atiomo *et al.*, 2009 | Low | Unclear | Low | Low | Low | Low | Low |
| Azadi-Yazdi *et al.*, 2017 | Low | Unclear | Low | Low | Low | Low | Low |
| Foroozanfard *et al.*, 2017 | Low | Low | Low | Low | Low | Low | Low |
| Gower *et al.*, 2013 and Goss *et al*., 2014 | Low | Unclear | Low | Unclear | Low | Low | Low |
| Kazemi *et al.*, 2018 and 2020 | Low | Low | Low | Low | Low | Low | Low |
| Mehrabani *et al.*, 2012 | Unclear | Low | Low | Low | High | Low | Low |
| Panico *et al*., 2014 | Unclear | Unclear | Unclear | Low | High | Low | Low |
| Turner-McGrievey *et al.*, 2014 | Low | High | High | Unclear | Low | Low | Low |
| Heather et al,2009 | Low | Unclear | Unclear | Unclear | Unclear | Low | Low |
| Manzoor et al, 2022 | Low | Unclear | Unclear | Unclear | Unclear | Low | Low |
| Pan et al, 2023 | Low | Unclear | Unclear | Unclear | Unclear | Low | Low |
| Hoover et al 2021 | Low | Low | Low | Unclear | Unclear | Low | Low |

**Supplementary Table 2 . Subgroup analysis of high/low GI and fiber diets in women with PCOS.**

| **Subgrouped by** | **Number of trials** | **Effect size** | **95% CI** | **P value for effect estimates** | **I^2^** | **P value for within-subgroup heterogeneity** | **P value for between-subgroup heterogeneity** |
| --- | --- | --- | --- | --- | --- | --- | --- |
| ***GI*** |  |  |  |  |  |  |  |
| **Fasting glucose** |  |  |  |  |  |  |  |
| Participant’s age |  |  |  |  |  |  | 0.61 |
| >30 y | 2 | -0.24 | -0.76, 0.27 | 0.36 | 75 | 0.04 |  |
| ≤30 y | 3 | -0.09 | -0.39, 0.21 | 0.57 | 29 | 0.24 |  |
| Energy restriction |  |  |  |  |  |  | 0.69 |
| Yes | 3 | -0.18 | -0.54, 0.18 | 0.33 | 42 | 0.18 |  |
| No | 2 | -0.07 | -0.45, 0.30 | 0.70 | 71 | 0.06 |  |
| Study duration |  |  |  |  |  |  | 0.02 |
| ≥16 wk | 2 | 0.34 | -0.13, 0.81 | 0.15 | 4 | 0.31 |  |
| <16 wk | 3 | -0.34 | -0.65, -0.02 | 0.03 | 0.0 | 0.79 |  |
| **Fasting insulin** |  |  |  |  |  |  |  |
| Participant’s age |  |  |  |  |  |  | 0.30 |
| >30 y | 2 | -0.10 | -0.61, 0.40 | 0.69 | 0.0 | 0.90 |  |
| ≤30 y | 3 | -0.42 | -0.72, -0.11 | 0.01 | 8 | 0.34 |  |
| Energy restriction |  |  |  |  |  |  | 0.11 |
| Yes | 3 | -0.54 | -0.91, -0.17 | 0.004 | 0.0 | 0.67 |  |
| No | 2 | -0.12 | -0.49, 0.26 | 0.54 | 0.0 | 1 |  |
| Study duration |  |  |  |  |  |  | 0.24 |
| ≥16 wk | 2 | -0.11 | -0.57, 0.36 | 0.66 | 0.0 | 0.90 |  |
| <16 wk | 3 | -0.44 | -0.76, -0.12 | 0.007 | 0 | 0.39 |  |
| **HOMA-IR** |  |  |  |  |  |  |  |
| Energy restriction |  |  |  |  |  |  | 0.03 |
| Yes | 2 | -0.59 | -0.97, -0.20 | 0.003 | 0.0 | 0.61 |  |
| No | 2 | 0.00 | -0.37, 0.37 | 1 | 0.0 | 0.42 |  |
| **TC** |  |  |  |  |  |  |  |
| Participant’s age |  |  |  |  |  |  | 0.96 |
| >30 y | 2 | -0.48 | -0.99, 0.03 | 0.07 | 0.0 | 0.59 |  |
| ≤30 y | 2 | -0.46 | -0.84, -0.08 | 0.02 | 0.0 | 0.45 |  |
| Energy restriction |  |  |  |  |  |  | 0.37 |
| Yes | 2 | -0.28 | -0.79, 0.24 | 0.29 | 0.0 | 0.87 |  |
| No | 2 | -0.57 | -0.95, -0.19 | 0.003 | 0.0 | 0.90 |  |
| Study duration |  |  |  |  |  |  | 0.73 |
| ≥16 wk | 2 | -0.53 | -1.00, -0.06 | 0.03 | 0.0 | 0.54 |  |
| <16 wk | 2 | -0.42 | -0.82, -0.02 | 0.04 | 0.0 | 0.55 |  |
| **LDL-C** |  |  |  |  |  |  |  |
| Participant’s age |  |  |  |  |  |  | 0.25 |
| >30 y | 2 | -0.57 | -1.09, -0.05 | 0.03 | 0.0 | 0.63 |  |
| ≤30 y | 2 | -0.19 | -0.57, 0.19 | 0.32 | 0.0 | 0.75 |  |
| Energy restriction |  |  |  |  |  |  | 0.71 |
| Yes | 2 | -0.24 | -0.76, 0.27 | 0.36 | 7 | 0.30 |  |
| No | 2 | -0.36 | -0.74, 0.01 | 0.06 | 0.0 | 0.49 |  |
| Study duration |  |  |  |  |  |  | 0.97 |
| ≥16 wk | 2 | -0.33 | -0.80, 0.14 | 0.17 | 0.0 | 0.38 |  |
| <16 wk | 2 | -0.32 | -0.72, 0.08 | 0.12 | 0.0 | 0.34 |  |
| **HDL-C** |  |  |  |  |  |  |  |
| Participant’s age |  |  |  |  |  |  | 0.31 |
| >30 y | 2 | -0.06 | -0.57, 0.44 | 0.81 | 0 | 0.34 |  |
| ≤30 y | 2 | 0.50 | -0.47, 1.47 | 0.31 | 84 | 0.01 |  |
| Energy restriction |  |  |  |  |  |  | 0.24 |
| Yes | 2 | -0.11 | -0.62, 0.41 | 0.68 | 0.0 | 0.38 |  |
| No | 2 | 0.52 | -0.40, 1.44 | 0.27 | 82 | 0.02 |  |
| Study duration |  |  |  |  |  |  | 0.75 |
| ≥16 wk | 2 | 0.29 | -1.27, 1.84 | 0.72 | 82 | 0.02 |  |
| <16 wk | 2 | 0.03 | -0.37, 0.42 | 0.90 | 0.0 | 0.90 |  |
| **TG** |  |  |  |  |  |  |  |
| Participant’s age |  |  |  |  |  |  | 0.14 |
| >30 y | 2 | -0.09 | -0.59, 0.42 | 0.73 | 0.0 | 0.78 |  |
| ≤30 y | 2 | -0.56 | -0.95, -0.18 | 0.004 | 32 | 0.22 |  |
| Energy restriction |  |  |  |  |  |  | 0.13 |
| Yes | 2 | -0.72 | -1.25, -0.19 | 0.008 | 0.0 | 0.38 |  |
| No | 2 | -0.22 | -0.59, 0.15 | 0.25 | 0.0 | 0.43 |  |
| Study duration |  |  |  |  |  |  | 0.79 |
| ≥16 wk | 2 | -0.34 | -0.81, 0.13 | 0.15 | 0.0 | 0.86 |  |
| <16 wk | 2 | -0.42 | -0.83, -0.02 | 0.04 | 72 | 0.06 |  |
| **Weight** |  |  |  |  |  |  |  |
| Participant’s age |  |  |  |  |  |  | 0.79 |
| >30 y | 3 | -0.35 | -0.75, 0.05 | 0.09 | 11 | 0.32 |  |
| ≤30 y | 4 | -0.49 | -1.45, 0.47 | 0.32 | 89 | <0.00001 |  |
| Energy restriction |  |  |  |  |  |  | 0.16 |
| Yes | 5 | -0.65 | -1.41, 0.11 | 0.09 | 82 | =0.0002 |  |
| No | 2 | -0.04 | -0.41, 0.33 | 0.83 | 0.0 | 0.96 |  |
| Study duration |  |  |  |  |  |  | 0.09 |
| ≥16 wk | 3 | 0.07 | -0.62, 0.76 | 0.84 | 48 | 0.15 |  |
| <16 wk | 4 | -0.78 | -1.49, -0.07 | 0.03 | 84 | 0.0004 |  |
| **WC** |  |  |  |  |  |  |  |
| Participant’s age |  |  |  |  |  |  | 0.85 |
| >30 y | 2 | -0.49 | -0.99, 0.01 | 0.06 | 58 | 0.12 |  |
| ≤30 y | 2 | -0.55 | -0.94, -0.17 | 0.005 | 63 | 0.10 |  |
| Study duration |  |  |  |  |  |  | 0.56 |
| ≥16 wk | 2 | -0.42 | -0.90, 0.06 | 0.08 | 63 | 0.10 |  |
| <16 wk | 2 | -0.60 | -1.00, -0.21 | 0.003 | 52 | 0.15 |  |
| **TT** |  |  |  |  |  |  |  |
| Participant’s age |  |  |  |  |  |  | 0.47 |
| >30 y | 3 | -0.53 | -1.26, 0.20 | 0.16 | 68 | 0.05 |  |
| ≤30 y | 2 | -0.21 | -0.64, 0.21 | 0.32 | 30 | 0.23 |  |
| Energy restriction |  |  |  |  |  |  | 0.41 |
| Yes | 3 | -0.54 | -1.25, 0.17 | 0.14 | 68 | 0.04 |  |
| No | 2 | -0.19 | -0.60, 0.22 | 0.36 | 17 | 0.27 |  |
| Study duration |  |  |  |  |  |  | 0.03 |
| ≥16 wk | 2 | 0.06 | -0.41, 0.52 | 0.81 | 0.0 | 0.57 |  |
| <16 wk | 3 | -0.66 | -1.11, -0.20 | 0.005 | 51 | 0.13 |  |
| **FAI** |  |  |  |  |  |  |  |
| Participant’s age |  |  |  |  |  |  | 0.44 |
| >30 y | 2 | -0.48 | -0.87, -0.09 | 0.02 | 66 | 0.09 |  |
| ≤30 y | 2 | -0.27 | -0.63, 0.09 | 0.15 | 55 | 0.13 |  |
| Energy restriction |  |  |  |  |  |  | 0.02 |
| Yes | 2 | -0.68 | -1.05, -0.30 | 0.0004 | 0.0 | 0.49 |  |
| No | 2 | -0.06 | -0.43, 0.31 | 0.76 | 0.0 | 0.73 |  |
| **SHGB** |  |  |  |  |  |  |  |
| Participant’s age |  |  |  |  |  |  | 0.40 |
| >30 y | 3 | 0.31 | -0.06, 0.68 | 0.10 | 69 | 0.04 |  |
| ≤30 y | 2 | 0.53 | 0.17, 0.89 | 0.004 | 0.0 | 0.52 |  |
| Energy restriction |  |  |  |  |  |  | 0.05 |
| Yes | 3 | 0.67 | 0.31, 1.03 | 0.0003 | 0.0 | 0.50 |  |
| No | 2 | 0.15 | -0.22, 0.53 | 0.43 | 56 | 0.13 |  |
| Study duration |  |  |  |  |  |  | 0.75 |
| ≥16 wk | 2 | 0.36 | -0.11, 0.83 | 0.13 | 0.0 | 0.58 |  |
| <16 wk | 3 | 0.45 | 0.14, 0.76 | 0.005 | 72 | 0.03 |  |
| ***FIber*** |  |  |  |  |  |  |  |
| **TT** |  |  |  |  |  |  |  |
| Participant’s age |  |  |  |  |  |  | 0.70 |
| >30 y | 2 | -0.48 | -1.76, 0.80 | 0.46 | 90 | 0.001 |  |
| ≤30 y | 2 | -0.20 | -0.76, 0.35 | 0.48 | 40 | 0.20 |  |
| **FAI** |  |  |  |  |  |  |  |
| Participant’s age |  |  |  |  |  |  | 0.34 |
| >30 y | 2 | -0.66 | -1.06, -0.27 | 0.0009 | 0.0 | 0.44 |  |
| ≤30 y | 2 | -0.39 | -0.81, 0.03 | 0.07 | 11 | 0.29 |  |
| **SHGB** |  |  |  |  |  |  |  |
| Participant’s age |  |  |  |  |  |  | 0.28 |
| >30 y | 2 | 0.85 | 0.45, 1.25 | ＜0.0001 | 0.0 | 0.92 |  |
| ≤30 y | 2 | 0.53 | 0.11, 0.95 | 0.01 | 0.0 | 0.44 |  |
| **Weight** |  |  |  |  |  |  |  |
| Participant’s age |  |  |  |  |  |  | 0.66 |
| >30 y | 2 | -0.46 | -0.85, -0.08 | 0.02 | 0.0 | 0.52 |  |
| ≤30 y | 5 | -0.64 | -1.32, 0.04 | 0.07 | 80 | 0.0004 |  |

Abbreviations: GL, Glycemic Load; HOMA-IR, Homeostatic Model Assessment of Insulin Resistance; TC, Total Cholesterol; LDL-C, Low-Density Lipoprotein Cholesterol; HDL-C, High-Density Lipoprotein Cholesterol; TG, Triglycerides; WC, Waist Circumference; TT, Total Testosterone; FAI, Free Androgen Index; SHBG, Sex Hormone-Binding Globulin.

Supplementary Figure 1. Subgroup analysis of the effects of low-GI dietary patterns on insulin resistance and sex hormones in PCOS women. (a) Effect of energy restriction on HOMA-IR in LGI dietary pattern studies; (b) effect of different study durations on fasting blood glucose in LGI dietary pattern studies; (c) effect of different study durations on TT in LGI dietary pattern studies; (d) effect of energy restriction on FAI in LGI dietary pattern studies; (e) effect of different ages on SHGB in LGI dietary pattern studies; (f) effect of energy restriction on SHGB in LGI dietary pattern studies.

Supplementary Figure 2. Forest plot of the effects of high-fiber, LGI, and LGL dietary patterns on fasting insulin in PCOS women. (a) Effects of a high-fiber diet on fasting insulin; (b) effects of an LGI diet on fasting insulin; (c) effects of an LGL diet on fasting insulin.

Supplementary Figure 3. Subgroup analysis of the effects on HDL-C in LGI dietary pattern studies: (a) effects of age on HDL-C in LGI dietary pattern studies; (b) effects of energy restriction on HDL-C in LGI dietary pattern studies; (c) effects of study duration on HDL-C in LGI dietary pattern studies.

Supplementary Figure 4. Forest plot of the effects of high-fiber, LGI, and LGL dietary patterns on TC in PCOS women. (a) Effects of high-fiber dietary patterns on TC; (b) effects of LGI dietary patterns on TC; (c) effects of LGL dietary patterns on TC.

Supplementary Figure 5. Forest plot of the effects of LGI dietary patterns on LH and FSH in PCOS women. (a) Effects of LGI dietary patterns on LH; (b) effects of LGI dietary patterns on FSH.

Supplementary Figure 6. Subgroup analyses of the effect on body weight in high-fiber and LGI dietary pattern studies in women with PCOS. (a) Effects of age on body weight in high-fiber dietary pattern studies; (b) effects of different study durations on body weight in LGI dietary pattern studies.

**Supplementary Figure 1**


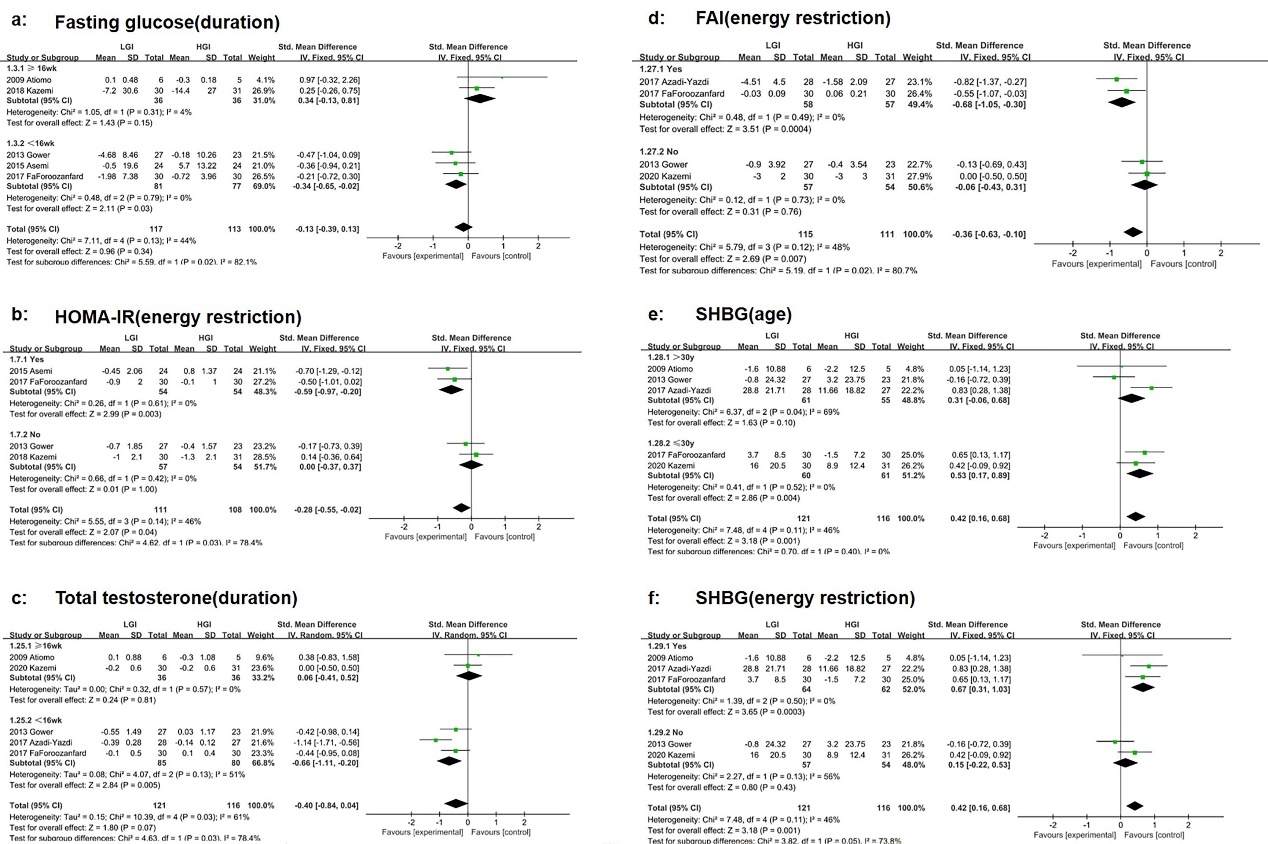


**Supplementary Figure 2**


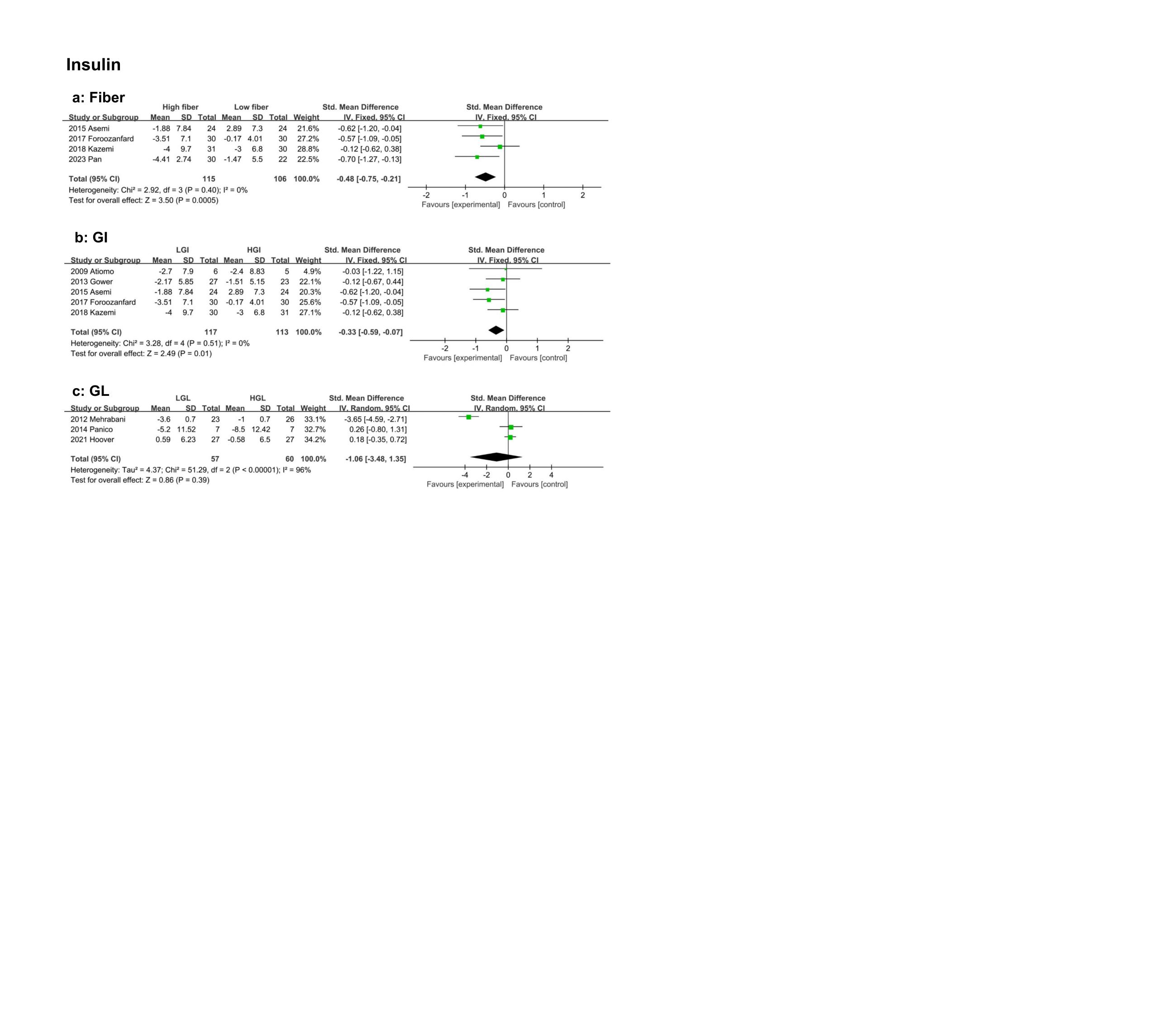


**Supplementary Figure 3**


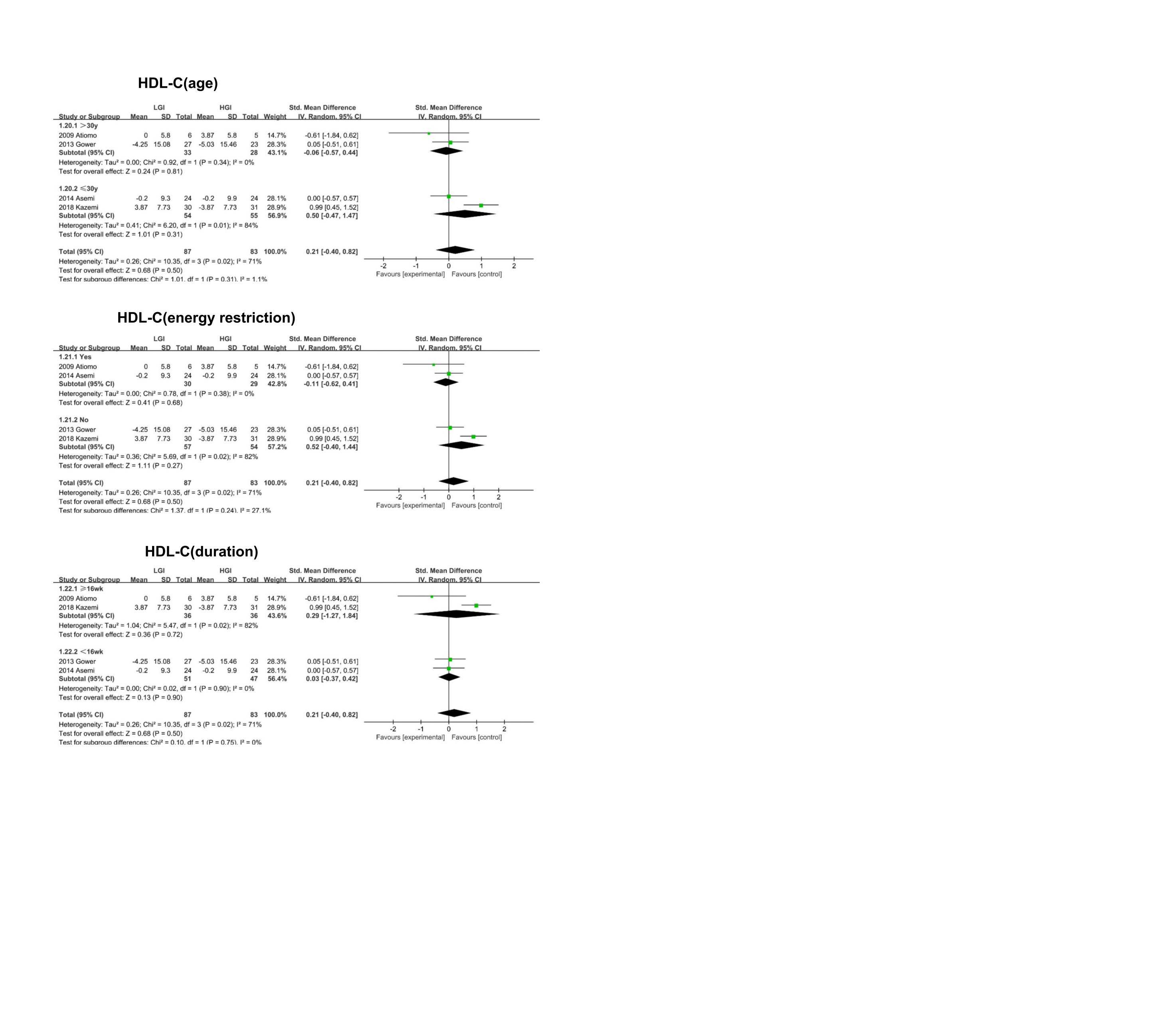


**Supplementary Figure 4**


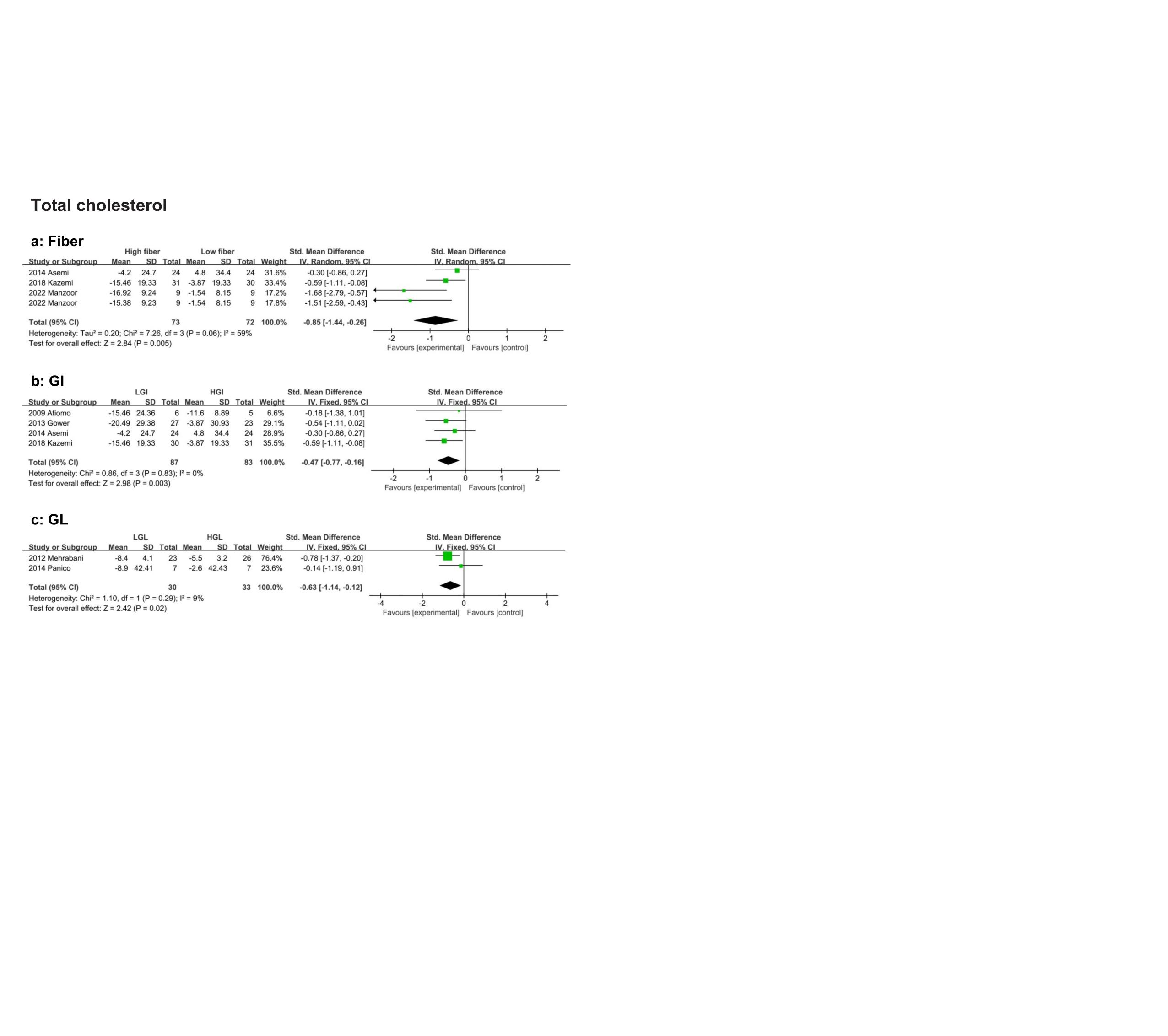


**Supplementary Figure 5**


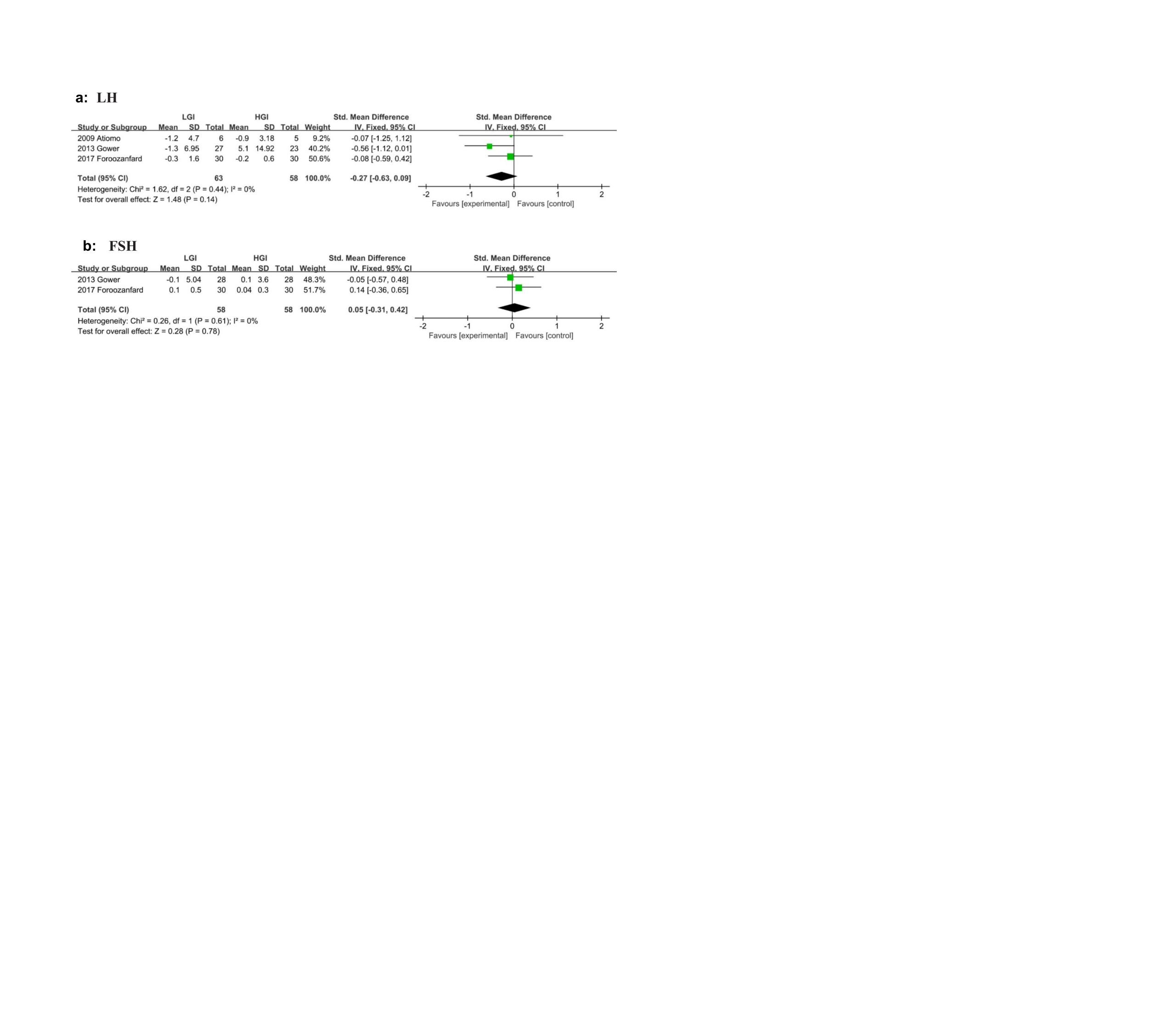


**Supplementary Figure 6**


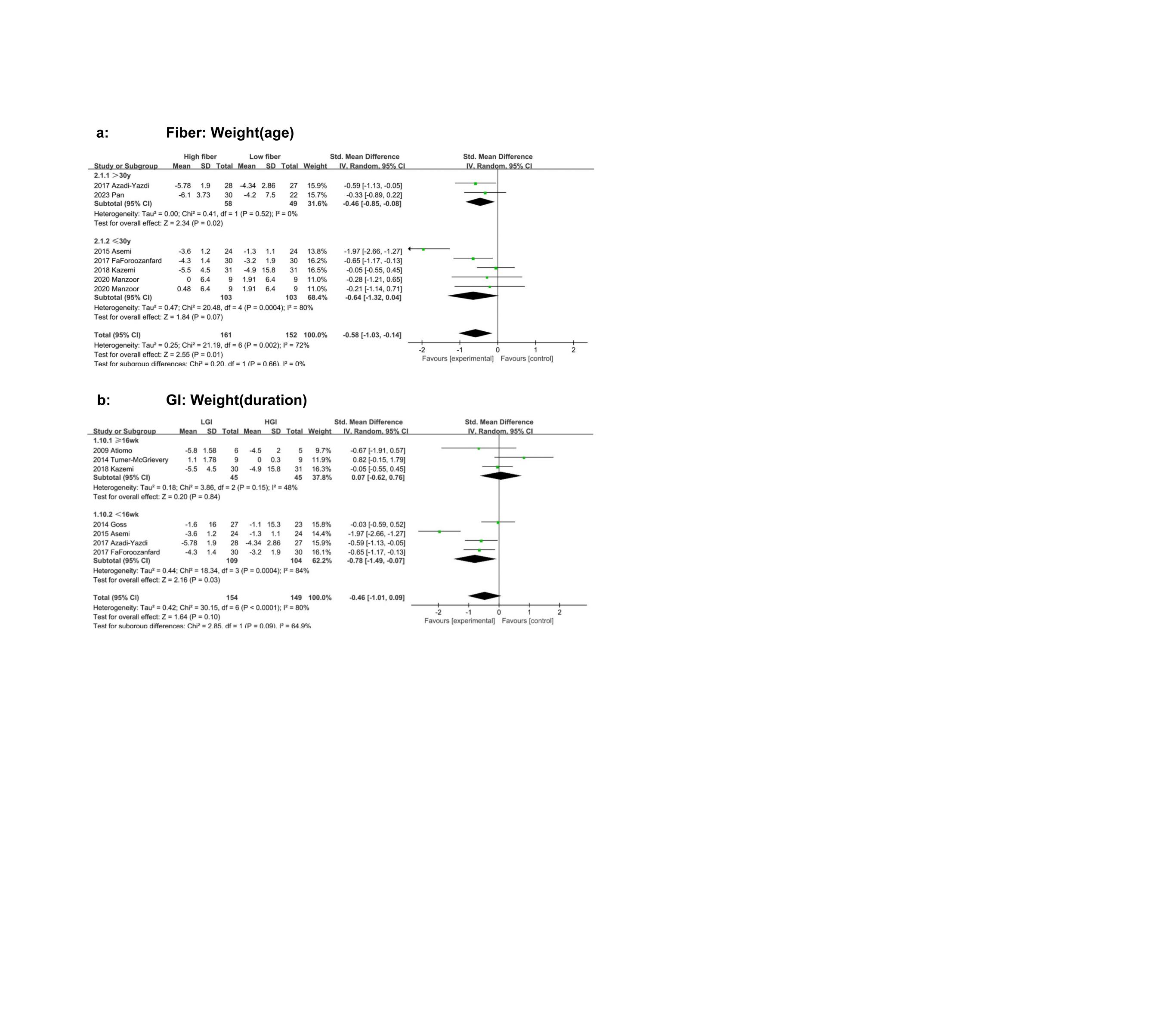


**Assessment of Publication Bias Using Begg's and Egger's tests**

1. **Publication bias. Begg and Egger tests of high-fiber diet on fasting glucose in PCOS women.**


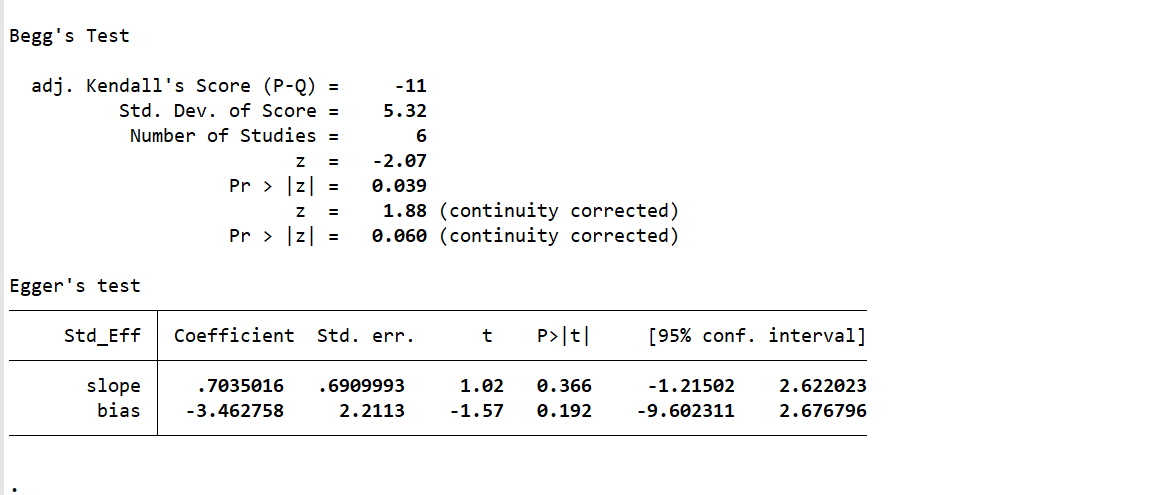


1. **Publication bias. Begg and Egger tests of LGI diet on HOMA-IR in PCOS women.**


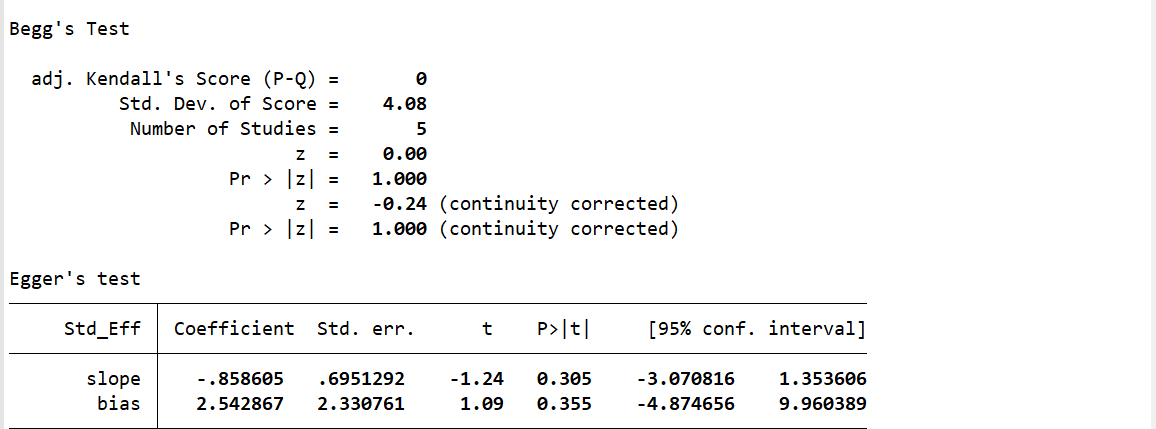


1. **Publication bias. Begg and Egger tests of LGI diet on fasting glucose in PCOS women.**


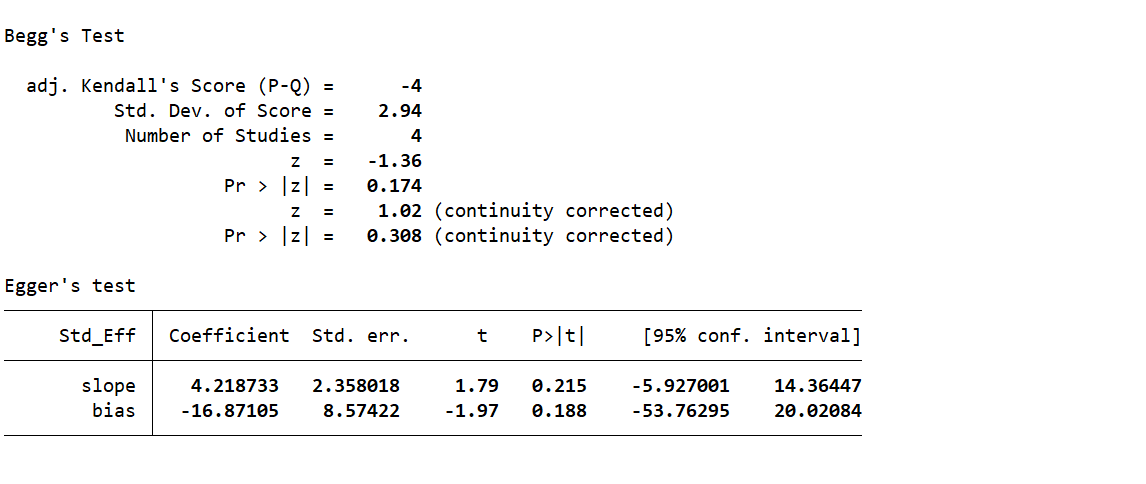


1. **Publication bias. Begg and Egger tests of high-fiber diet on HOMA-IR in PCOS women.**


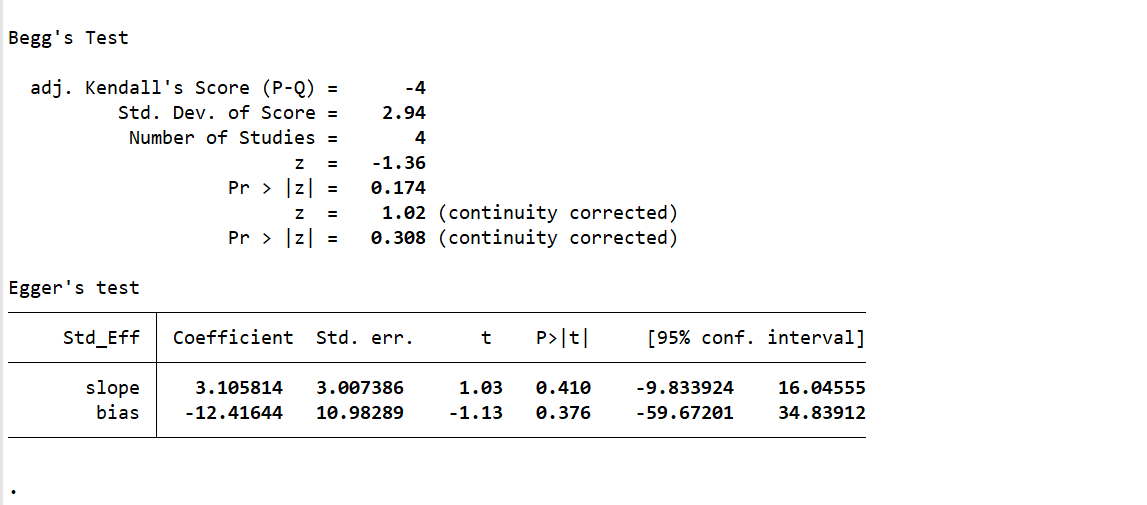


1. **Publication bias.Begg and Egger tests of high-fiber diet on LDL-C in PCOS women.**
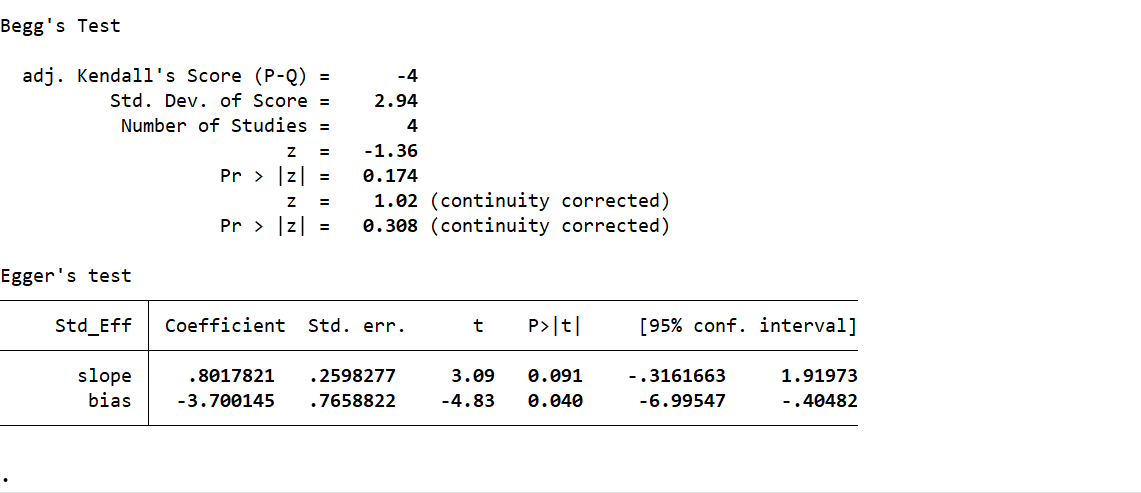


1. **Publication bias. Begg and Egger tests of LGI diet on LDL cholesterol in PCOS women.**


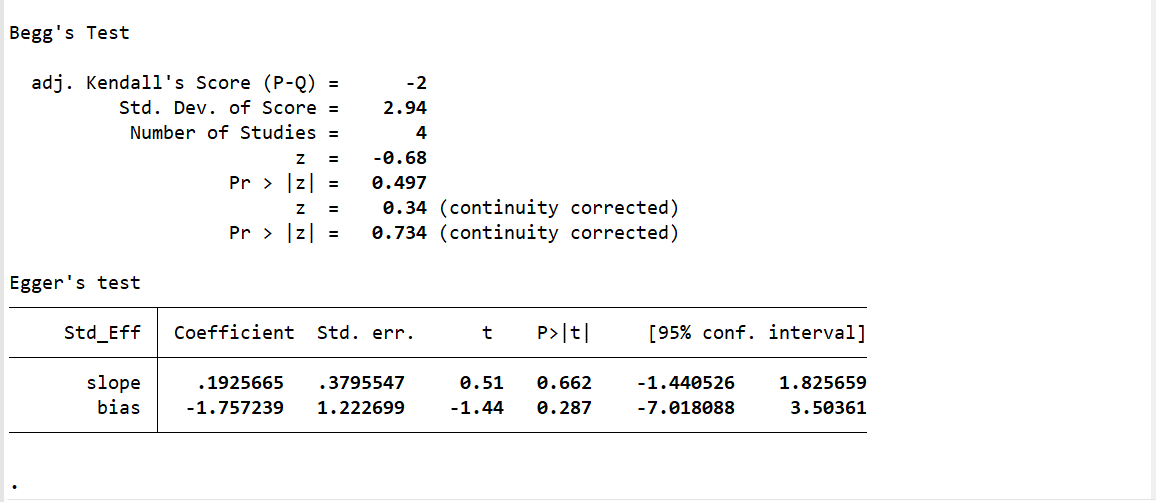


1. **Publication bias. Begg and Egger tests of high-fiber diet on HDL cholesterol in PCOS women.**


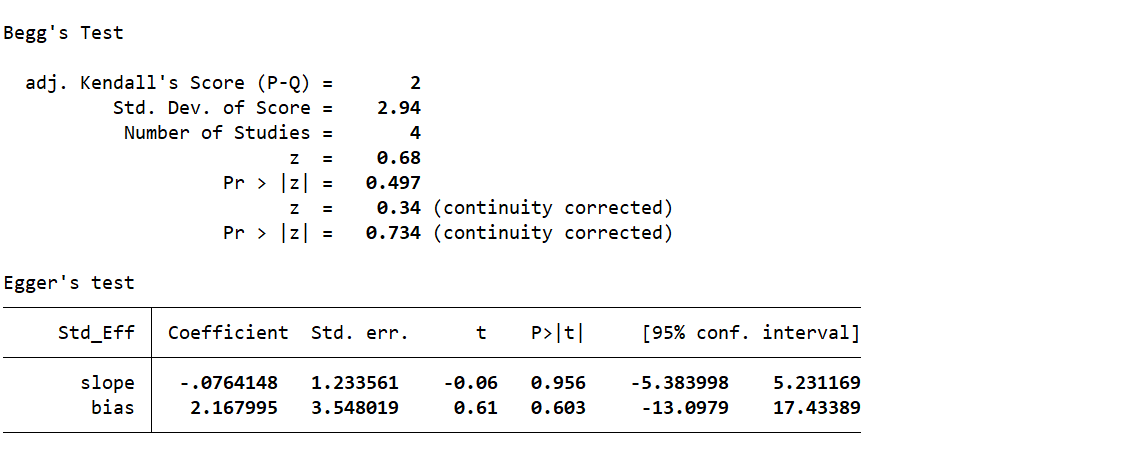


1. **Publication bias. Begg and Egger tests of LGI diet on HDL cholesterol in PCOS women.**

**
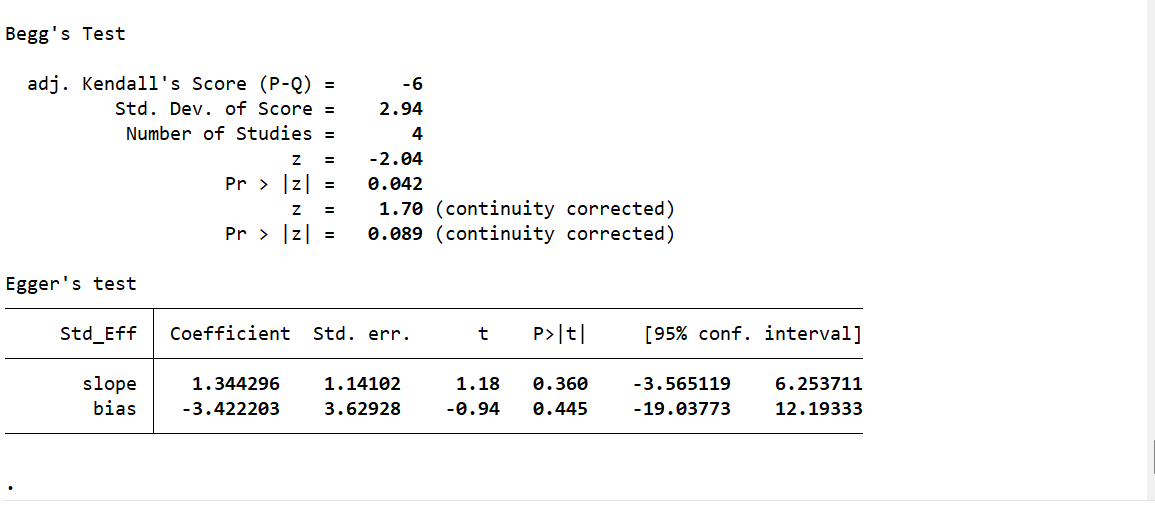
**

1. **Publication bias. Begg and Egger tests of high-fiber diet on triglycerides in PCOS women.**


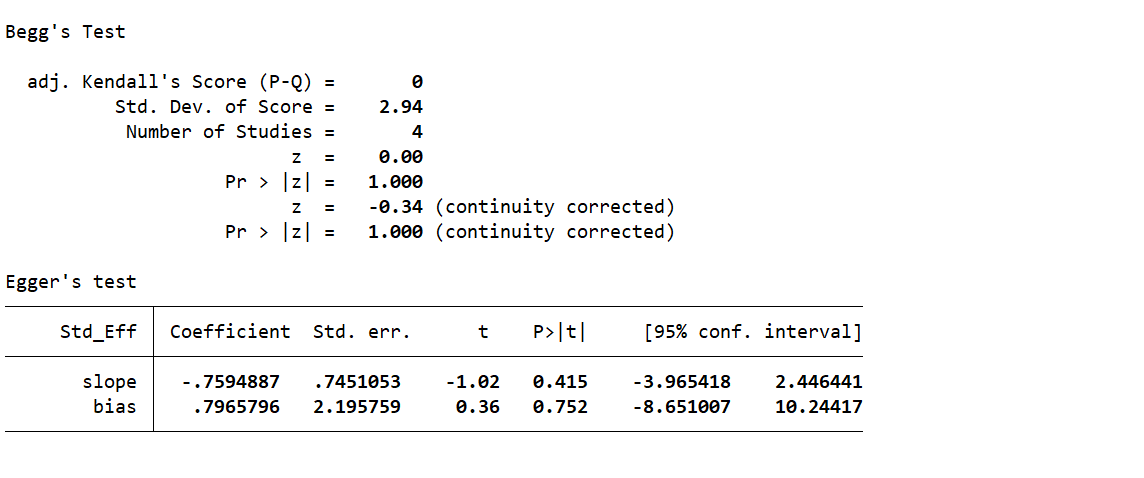


1. **Publication bias. Begg and Egger tests of low-GI diet on triglycerides in PCOS women.**


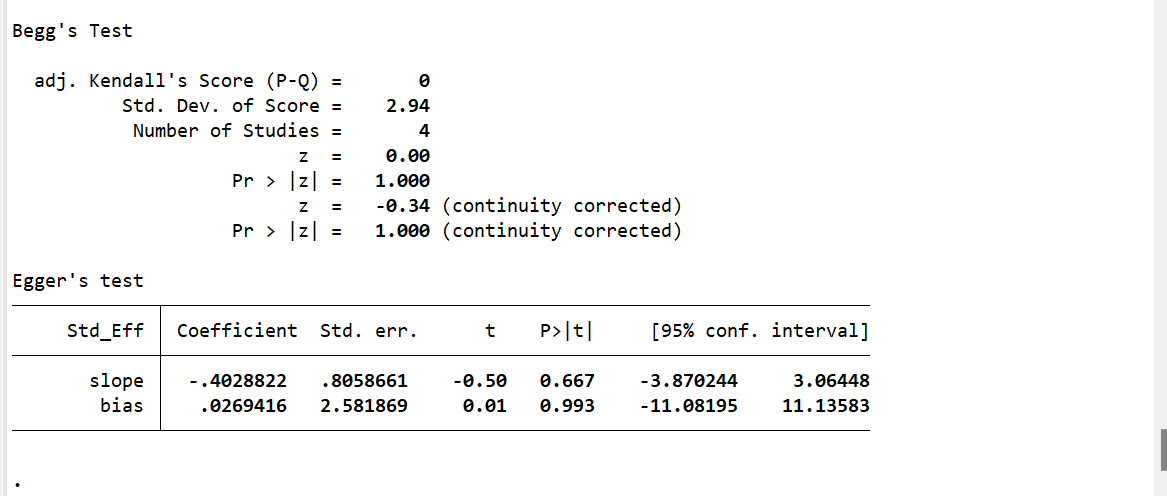


1. **Publication bias. Begg and Egger tests of high-fiber diet on total testosterone in PCOS women.**


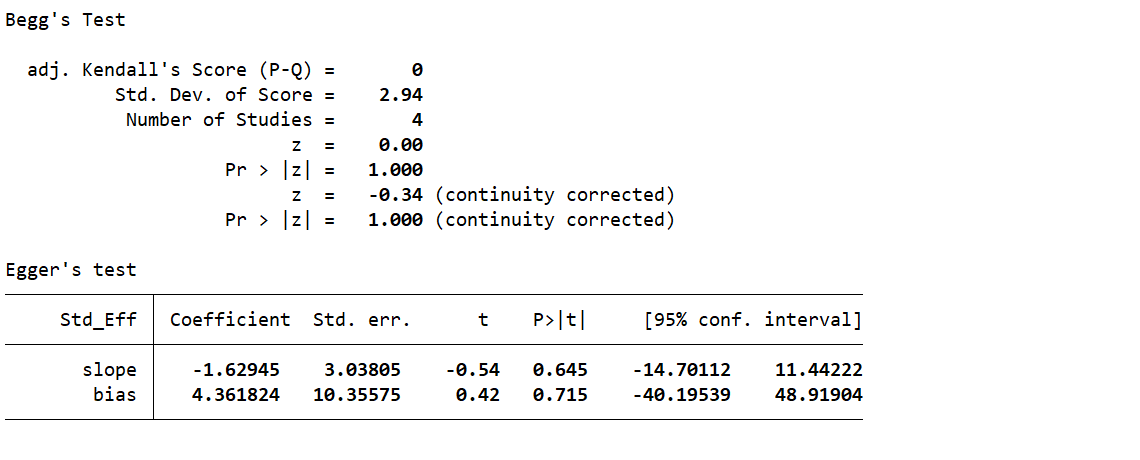


1. **Publication bias. Begg and Egger tests of low-GI diet on total testosterone in PCOS women.**


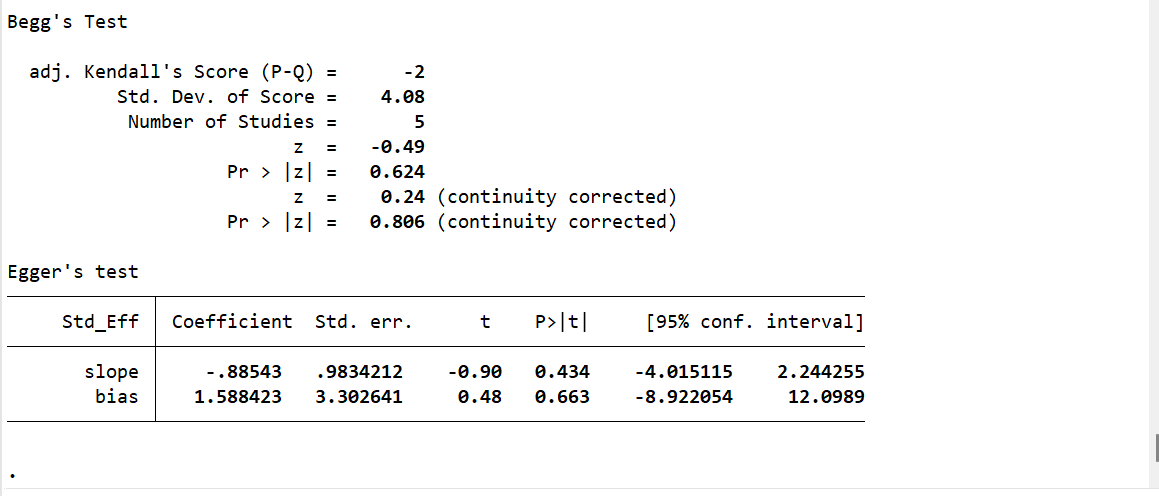


1. **Publication bias. Begg and Egger tests of high-fiber diet on FAI in PCOS women.**


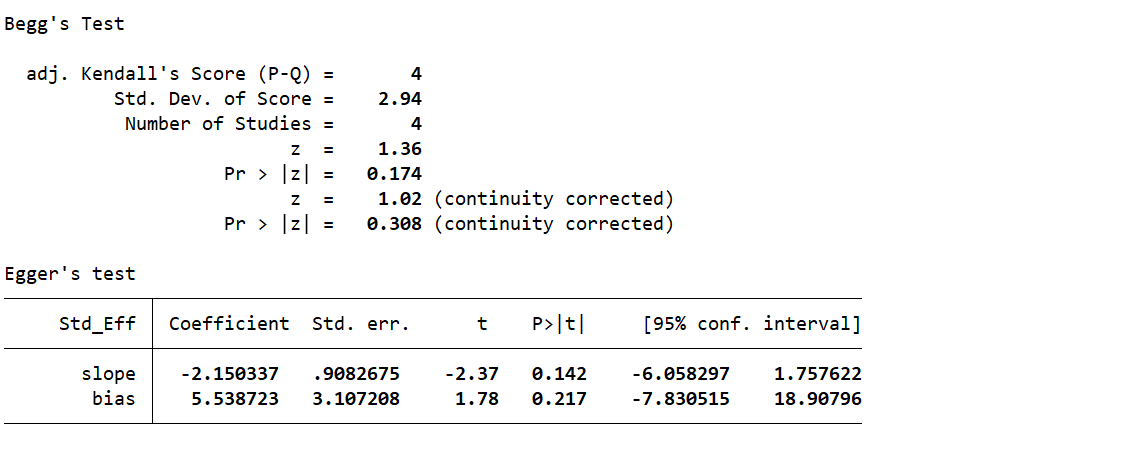


1. **Publication bias. Begg and Egger tests of low-GI diet on FAI in PCOS women.**


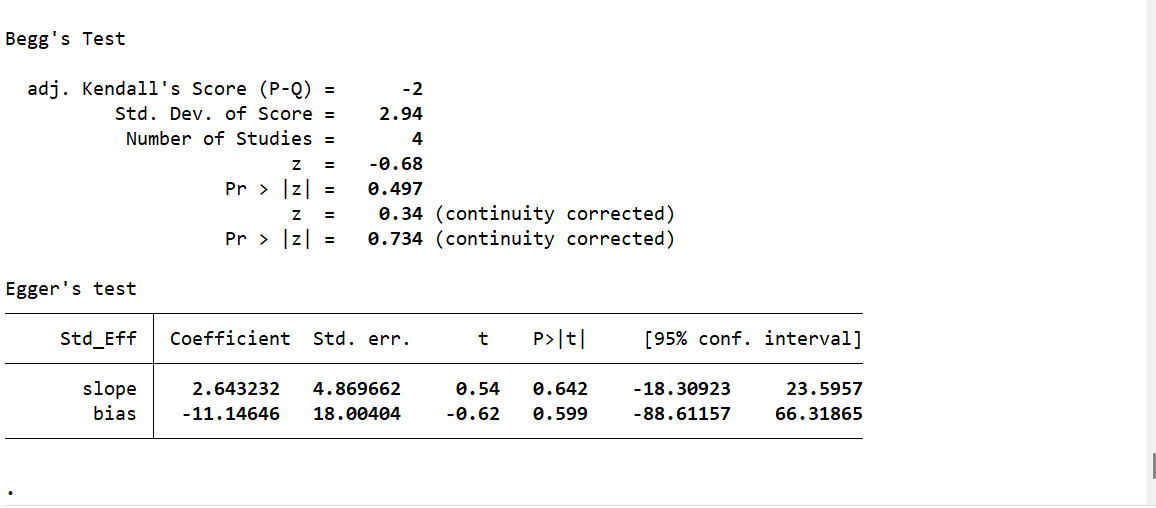


1. **Publication bias. Begg and Egger tests of high-fiber diet on SHBG in PCOS women.**


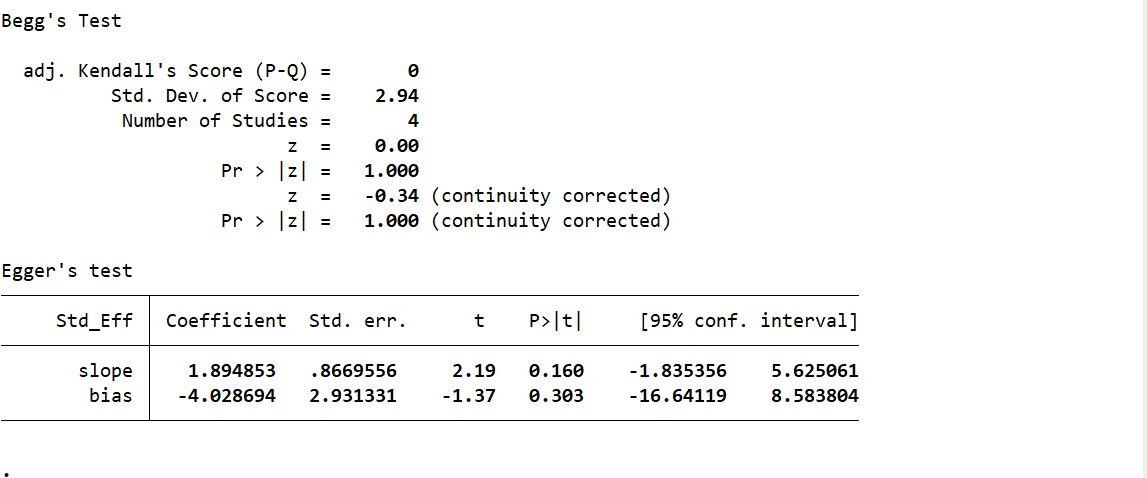


1. **Publication bias. Begg and Egger tests of low-GI diet on SHBG in PCOS women.**


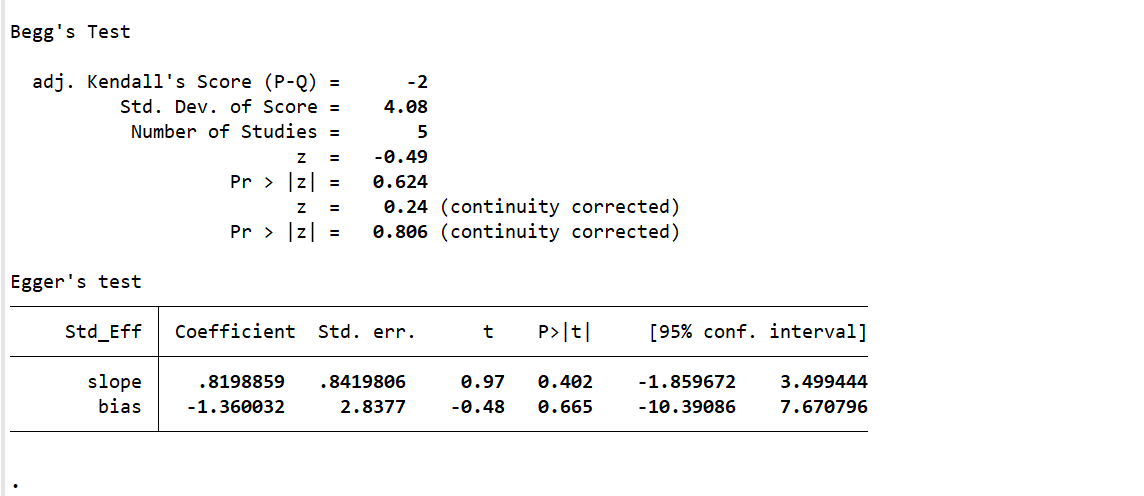


1. **Publication bias. Begg and Egger tests of high-fiber diet on weight in PCOS women.**


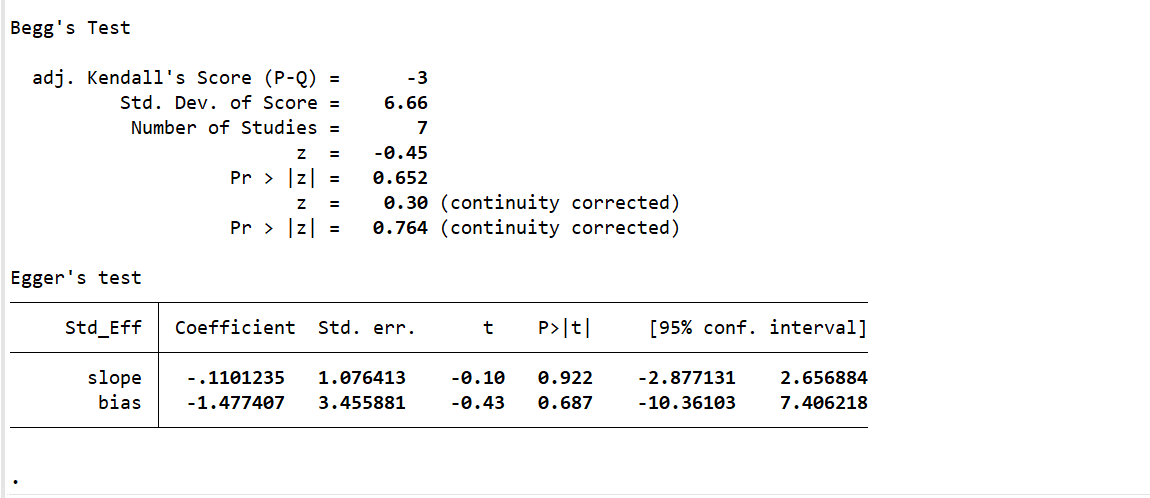


1. **Publication bias. Begg and Egger tests of low-GI diet on weight in PCOS women.**


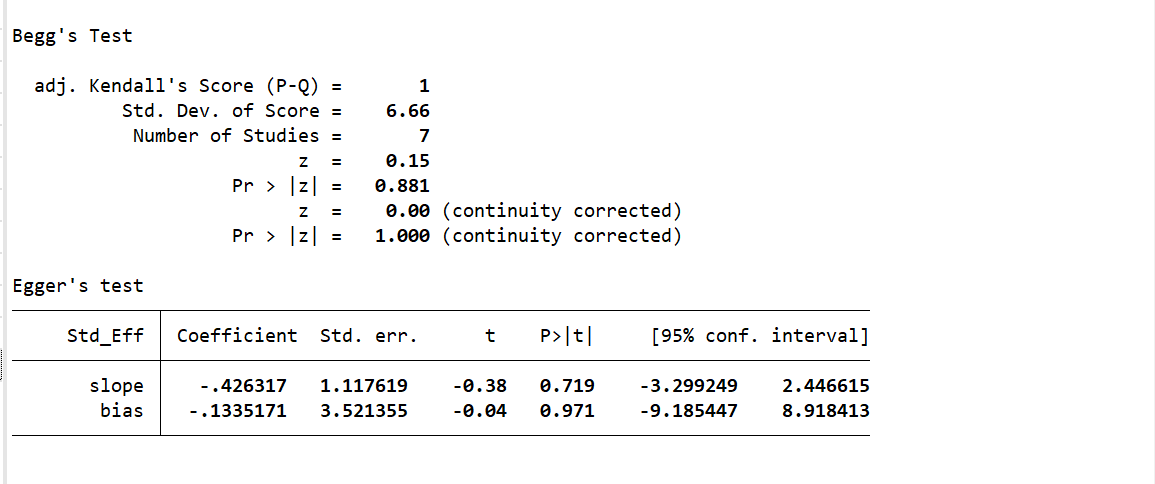


1. **Publication bias. Begg and Egger tests of low-GI diet on waist circumference in PCOS women.**


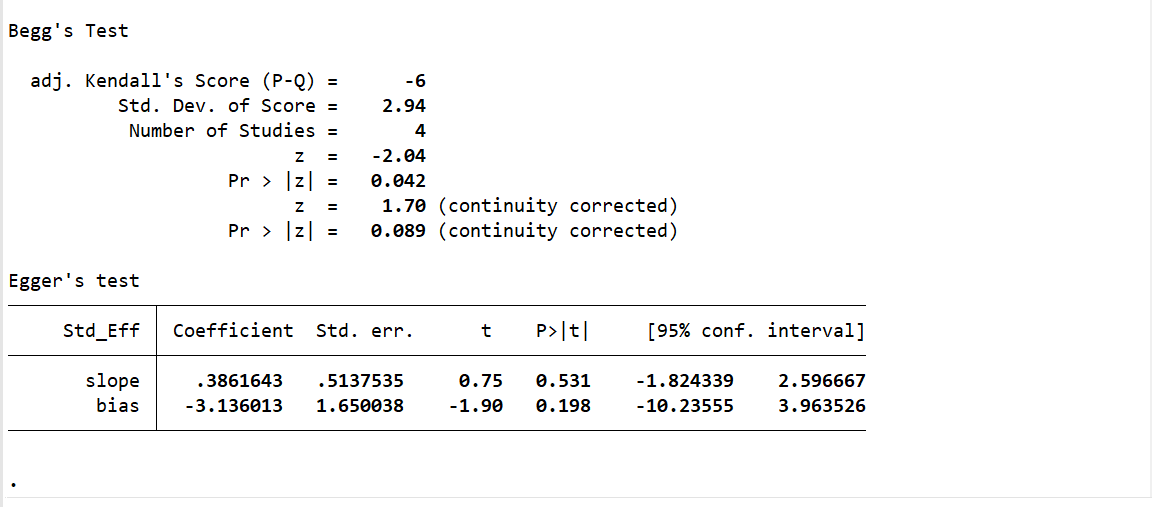


**Search strategy in Pubmed**

*Fiber*

#1 "Dietary Fiber"[Mesh]

#2 ((((Dietary Fibers [Title/Abstract]) OR (Wheat Bran[Title/Abstract])) OR (Wheat Brans[Title/Abstract])) OR (Roughage[Title/Abstract])) OR (Roughages[Title/Abstract])

#3 #1 OR #2

#4 (Dietary Carbohydrate[Title/Abstract]) OR ("Dietary Carbohydrates"[Mesh])

#5 #3 OR #4

#6 (((((((Ovary Syndrome Polycystic[Title/Abstract]) OR (Stein Leventhal Syndrome[Title/Abstract])) OR (Sclerocystic Ovarian Degeneration[Title/Abstract])) OR (Polycystic Ovarian Syndrome[Title/Abstract])) OR (Polycystic Ovary Syndrome 1[Title/Abstract])) OR (Sclerocystic Ovaries[Title/Abstract])) OR (Sclerocystic Ovary[Title/Abstract])) OR ("Polycystic Ovary Syndrome"[Mesh])

#7 #5 AND #6

*GI & GL*

#1 ("Glycemic Load"[Mesh]) OR (Glycemic Load[Title/Abstract])

#2 ((("Glycemic Index"[Mesh]) OR (Glycemic Indices[Title/Abstract])) OR (Glycemic Index Number[Title/Abstract])) OR (Glycemic Index Numbers[Title/Abstract])

#3 #1 OR #2

#4 (((((((Ovary Syndrome Polycystic[Title/Abstract]) OR (Stein Leventhal Syndrome[Title/Abstract])) OR (Sclerocystic Ovarian Degeneration[Title/Abstract])) OR (Polycystic Ovarian Syndrome[Title/Abstract])) OR (Polycystic Ovary Syndrome 1[Title/Abstract])) OR (Sclerocystic Ovaries[Title/Abstract])) OR (Sclerocystic Ovary[Title/Abstract])) OR ("Polycystic Ovary Syndrome"[Mesh])

#5 #3 AND #4

*Whole grains and refined grains*

#1 "Whole Grains"[Mesh] OR "Refined Grains"[Mesh]

#2 ((Grain Whole[Title/Abstract]) OR (Whole Grain Cereals[Title/Abstract])) OR (Whole Grain Cereal[Title/Abstract]) OR ((Refined Whole[Title/Abstract]) OR (Refined Grain Cereals[Title/Abstract])) OR (Refined Grain Cereal[Title/Abstract])

#3 #1 OR #2

#4 "Polycystic Ovary Syndrome"[Mesh]

#5 ((((((Ovary Syndrome Polycystic[Title/Abstract]) OR (Stein Leventhal Syndrome[Title/Abstract])) OR (Sclerocystic Ovarian Degeneration[Title/Abstract])) OR (Polycystic Ovarian Syndrome[Title/Abstract])) OR (Polycystic Ovary Syndrome 1[Title/Abstract])) OR (Sclerocystic Ovaries[Title/Abstract])) OR (Sclerocystic Ovary[Title/Abstract])

#6 #4 OR #5

#7 "Dietary Carbohydrates"[Mesh]

#8 Dietary Carbohydrate[Title/Abstract]

#9 #7 OR #8

#10 #3 AND #6 AND #9

**Search strategy in EMBASE**

*Fiber*

#1 'ovary polycystic disease'/exp/mj OR 'cystic ovary':ab,ti OR 'micropolycystic ovary':ab,ti OR 'multiple follicle cyst':ab,ti OR 'ovary polycystic syndrome':ab,ti OR 'ovary, micropolycystic':ab,ti OR 'ovary, polycystic':ab,ti OR 'polycystic ovarian disease':ab,ti OR 'polycystic ovary':ab,ti OR 'polycystic ovary disease':ab,ti OR 'polycystic ovary syndrome':ab,ti OR 'stein cohen leventhal syndrome':ab,ti OR 'stein leventhal disease':ab,ti OR 'stein leventhal syndrome':ab,ti OR 'syndrome stein leventhal':ab,ti OR 'ovary polycystic disease':ab,ti

#2 'fiber intake'/exp OR 'fiber intake'

#3 'dietary fiber intake':ab,ti OR 'fiber consumption':ab,ti OR 'fiber intake':ab,ti

#4 #2 OR #3

#5 #1 AND #4

*GI & GL*

#1 'glycemic index'/exp OR 'glycemic index'

#2 'glycaemia index':ab,ti OR 'glycaemic value':ab,ti OR 'glycemia index':ab,ti OR 'glycemic value':ab,ti OR 'glycemie index':ab,ti OR 'glycemic index':ab,ti

#3 'glycemic load'/exp OR 'glycemic load'

#4 'glycemic load':ab,ti

#5 #1 OR #2 OR #3 OR #4

#6 'ovary polycystic disease'/exp OR 'ovary polycystic disease'

#7 'cystic ovary':ab,ti OR 'micropolycystic ovary':ab,ti OR 'multiple follicle cyst':ab,ti OR 'ovary polycystic syndrome':ab,ti OR 'ovary micropolycystic':ab,ti OR 'polycystic ovarian disease':ab,ti OR 'polycystic ovary':ab,ti OR 'polycystic ovary syndrome':ab,ti OR 'stein cohen leventhal syndrome':ab,ti OR 'stein leventhal':ab,ti

#8 #6 OR #7

#9 #5 AND #8

*Whole grains and refined grains*

#1 'food grain'/exp/mj OR 'edible grain':ab,ti OR 'whole grain'/exp/mj OR 'unrefined grain':ab,ti OR 'whole grains':ab,ti OR wholegrain:ab,ti OR wholegrains:ab,ti OR 'refined grain'/exp/mj

#2 'ovary polycystic disease'/exp/mj

#3 'cystic ovary':ab,ti OR 'micropolycystic ovary':ab,ti OR 'multiple follicle cyst':ab,ti OR 'ovary polycystic syndrome':ab,ti OR 'ovary micropolycystic':ab,ti OR 'polycystic ovarian disease':ab,ti OR 'polycystic ovary':ab,ti OR 'polycystic ovary syndrome':ab,ti OR 'stein cohen leventhal syndrome':ab,ti OR 'stein leventhal':ab,ti

#4 #2 OR #3

#5 #1 AND #4

#6 'carbohydrate intake'/exp/mj OR 'alimentary carbohydrate':ab,ti OR 'carbohydrate consumption':ab,ti OR 'carbohydrate feeding':ab,ti OR 'diet carbohydrate':ab,ti OR 'dietary carbohydrate':ab,ti OR 'dietary carbohydrate intake':ab,ti OR 'dietary carbohydrates':ab,ti OR 'saccharide intake':ab,ti OR 'carbohydrate intake':ab,ti

#7 #1 OR #6

#8 #4 AND #7

**Search strategy associated in the Cochrane Library**

*Fiber*

#1 (Polycystic Ovary Syndrome):ti,ab,kw OR (Stein Leventhal Syndrome):ti,ab,kw OR (Ovarian Degeneration, Sclerocystic):ti,ab,kw OR ("sclerocystic ovaries"):ti,ab,kw

#2 (dietary fiber):ti,ab,kw OR (dietary Fibers):ti,ab,kw OR (Wheat Bran):ti,ab,kw OR (Wheat Brans):ti,ab,kw OR (Roughage):ti,ab,kw

#3 (dietary Carbohydrates):ti,ab,kw OR (dietary carbohydrate):ti,ab,kw

#4 #2 OR #3

#5 #1 AND #4

*Whole grains and refined grains*

#1 (Polycystic Ovary Syndrome):ti,ab,kw OR (Stein Leventhal Syndrome):ti,ab,kw OR (Ovarian Degeneration, Sclerocystic):ti,ab,kw OR ("sclerocystic ovaries"):ti,ab,kw

#2 (whole grain):ti,ab,kw OR (Whole Grains):ti,ab,kw OR (Whole Grain Cereal):ti,ab,kw OR (Whole Grain Cereals):ti,ab,kw OR (Refined Grain):ti,ab,kw

#3 (dietary Carbohydrates):ti,ab,kw OR (dietary carbohydrate):ti,ab,kw

#4 #2 OR #3

#5 #1 AND #4

**Search strategy in Web of Scienc**

*Fiber*

(TS=("Dietary Fiber" or "Dietary Fibers" or "Wheat Bran" or "Wheat Brans" or "Roughage" or "Roughages")) AND TS=("Ovary Syndrome Polycystic" OR "Stein Leventhal Syndrome" OR "Sclerocystic Ovarian Degeneration" OR "Polycystic Ovarian Syndrome" OR "Polycystic Ovary Syndrome 1" OR "Sclerocystic Ovaries" OR "Sclerocystic Ovary" OR "Polycystic Ovary Syndrome")

*GI & GL*

((TS=("Ovary Syndrome Polycystic" OR "Stein Leventhal Syndrome" OR "Sclerocystic Ovarian Degeneration" OR "Polycystic Ovarian Syndrome" OR "Polycystic Ovary Syndrome 1" OR "Sclerocystic Ovaries"OR "Sclerocystic Ovary" OR "Polycystic Ovary Syndrome")) AND TS=("Glycemic Index" OR "Glycemic Indices" OR "Glycemic Index Number"OR "Glycemic Index Numbers" OR "Glycemic Load" OR "Glycemic Load"))

*Whole grains and refined grains*

#1 TS=("Ovary Syndrome Polycystic" OR "Stein Leventhal Syndrome" OR "Sclerocystic Ovarian Degeneration" OR "Polycystic Ovarian Syndrome" OR "Polycystic Ovary Syndrome 1" OR "Sclerocystic Ovaries"OR "Sclerocystic Ovary" OR "Polycystic Ovary Syndrome"

#2 TS=("Grain Whole"OR "Whole Grain Cereals" OR "Whole Grain Cereal"OR "Whole Grains")) OR TS=("Dietary Carbohydrate" OR "Dietary Carbohydrates")

#3 TS=("Refined Grain" OR " Refined Grain Cereals" OR " Refined Grain Cereal"OR " Refined Grains")) OR TS=("Dietary Carbohydrate" OR "Dietary Carbohydrates")

#4 #2 OR #3

#5 #1 AND #4

**Search strategy in Ovid MEDLINE**

*Fiber*

#1 (Dietary Fiber OR Wheat Bran OR Wheat Brans OR Roughage OR Roughages OR Dietary Fibers OR Dietary Carbohydrate OR Dietary Carbohydrates).ti,ab,kw

#2 (Polycystic Ovary Syndrome OR Ovary Syndrome Polycystic OR Stein Leventhal Syndrome OR Sclerocystic Ovarian Degeneration OR Polycystic Ovarian Syndrome OR Sclerocystic Ovaries).ti,ab,kw

#3 #1 AND #2

*GI & GL*

#1 (Glycemic Load OR OR Glycemic Indices OR Glycemic Index Number OR Glycemic Index Numbers).ti,ab,kw

#2 (Polycystic Ovary Syndrome OR Ovary Syndrome Polycystic OR Stein Leventhal Syndrome OR Sclerocystic Ovarian Degeneration OR Polycystic Ovarian Syndrome OR Sclerocystic Ovaries).ti,ab,kw

#3 #1 AND #2

*Whole grains and refined grains*

#1 (Whole Grains OR Grain Whole OR Whole Grain Cereals OR Whole Grain Cereal OR Dietary Carbohydrate OR Dietary Carbohydrates).ti,ab,kw

#2 (Refined Grains OR Grain Refined OR Refined Grain Cereals OR Refined Grain Cereal OR Refined Grain).ti,ab,kw

#3 #1 OR #2

#4 (Polycystic Ovary Syndrome OR Ovary Syndrome Polycystic OR Stein Leventhal Syndrome OR Sclerocystic Ovarian Degeneration OR Polycystic Ovarian Syndrome OR Sclerocystic Ovaries).ti,ab,kw

#5 #3 AND #4

**Search strategy in Scopus**

*Fiber*

#1 TITLE-ABS-KEY (Dietary Fiber OR Wheat Bran OR Wheat Brans OR Roughage OR Roughages OR Dietary Fibers OR Dietary Carbohydrate OR Dietary Carbohydrates)

#2 TITLE-ABS-KEY (Polycystic Ovary Syndrome OR Ovary Syndrome Polycystic OR Stein Leventhal Syndrome OR Sclerocystic Ovarian Degeneration OR Polycystic Ovarian Syndrome OR Sclerocystic Ovaries)

#3 #1 AND #2

*GI & GL*

#1 TITLE-ABS-KEY (Glycemic Load OR OR Glycemic Indices OR Glycemic Index Number OR Glycemic Index Numbers)

#2 TITLE-ABS-KEY (Polycystic Ovary Syndrome OR Ovary Syndrome Polycystic OR Stein Leventhal Syndrome OR Sclerocystic Ovarian Degeneration OR Polycystic Ovarian Syndrome OR Sclerocystic Ovaries)

#3 #1 AND #2

*Whole grains and refined grains*

#1 TITLE-ABS-KEY (Whole Grains OR Grain Whole OR Whole Grain Cereals OR Whole Grain Cereal OR Refined Grains OR Grain Refined OR Refined Grain Cereals OR Refined Grain Cereal OR Refined Grain OR Dietary Carbohydrate OR Dietary Carbohydrates)

#2 TITLE-ABS-KEY (Polycystic Ovary Syndrome OR Ovary Syndrome Polycystic OR Stein Leventhal Syndrome OR Sclerocystic Ovarian Degeneration OR Polycystic Ovarian Syndrome OR Sclerocystic Ovaries)

#3 #1 AND #2
